# Unravelling the Viral Hypothesis of Schizophrenia: A Comprehensive Review of Mechanisms and Evidence

**DOI:** 10.3390/ijms26157429

**Published:** 2025-08-01

**Authors:** Mădălina Georgeta Sighencea, Simona Corina Trifu

**Affiliations:** 1 Doctoral School, “Carol Davila” University of Medicine and Pharmacy Bucharest, 020021 Bucharest, Romania; madalina-georgeta.iliescu@drd.umfcd.ro; 2 Department of Psychiatry, “Carol Davila” University of Medicine and Pharmacy Bucharest, 020021 Bucharest, Romania

**Keywords:** schizophrenia, viral infections, neuroinflammation, maternal immune activation, herpesviruses, viromics, immunogenetics

## Abstract

Schizophrenia is a challenging multifactorial neuropsychiatric disease that involves interactions between genetic susceptibility and environmental insults. Increasing evidence implicates viral infections as significant environmental contributors, particularly during sensitive neurodevelopmental periods. This review synthesises current findings on the viral hypothesis of schizophrenia, encompassing a wide array of neurotropic viruses, including influenza viruses, herpesviruses (HSV-1 and 2, CMV, VZV, EBV, HHV-6 and 8), hepatitis B and C viruses, HIV, HERVs, HTLV, Zika virus, BoDV, coronaviruses (including SARS-CoV-2), and others. These pathogens can contribute to schizophrenia through mechanisms such as direct microinvasion, persistent central nervous system infection, immune-mediated neuroinflammation, molecular mimicry, and the disturbance of the blood–brain barrier. Prenatal exposure to viral infections can trigger maternal immune activation, resulting in cytokine-mediated alterations in the neurological development of the foetus that persist into adulthood. Genetic studies highlight the role of immune-related loci, including major histocompatibility complex polymorphisms, in modulating susceptibility to infection and neurodevelopmental outcomes. Clinical data also support the “mild encephalitis” hypothesis, suggesting that a subset of schizophrenia cases involve low-grade chronic neuroinflammation. Although antipsychotics have some immunomodulatory effects, adjunctive anti-inflammatory therapies show promise, particularly in treatment-resistant cases. Despite compelling associations, pathogen-specific links remain inconsistent, emphasising the need for longitudinal studies and integrative approaches such as viromics to unravel causal relationships. This review supports a “multi-hit” model in which viral infections interfere with hereditary and immunological susceptibilities, enhancing schizophrenia risk. Elucidating these virus–immune–brain interactions may facilitate the discovery of biomarkers, targeted prevention, and novel therapeutic strategies for schizophrenia.

## 1. Introduction

Schizophrenia is a common debilitating neuropsychiatric disease, and contemporary research reveals a multifaceted aetiology that spans genetic, neurochemical, and environmental factors. Therefore, emerging evidence highlights the critical role of infectious agents, particularly neurotropic viruses, in disease pathogenesis. This viral hypothesis complements other infectious theories involving bacterial pathogens such as *Chlamydia* species and the protozoan parasite *Toxoplasma gondii*, collectively painting an image of schizophrenia as a disease shaped by microbial influences during vulnerable neurodevelopmental periods [1,2].

Therefore, *C. psittaci* and *C. pneumoniae* have been identified as relevant infectious risk factors in the pathogenesis of schizophrenia, acting through several converging mechanisms. *C. psittaci*, a neurotropic microorganism, may exert direct neurotoxic effects, whereas *C. pneumoniae* has been implicated in disrupting the blood–brain barrier. Both pathogens can infect monocytes that infiltrate the central nervous system, promoting microglial activation and sustained neuroinflammation. Additionally, these infections may alter cytokine signalling, particularly interleukin-2 (IL-2), leading to impaired production of neurotrophins such as brain-derived neurotrophic factor (BDNF) and neurotrophin-3 (NT-3), which are vital for neuronal development and synaptic plasticity. Genetic vulnerability further amplifies this risk, as carriers of the human leukocyte antigen-A10 (HLA-A10) allele show suboptimal immune responses to *C. psittaci*, indicating a gene–environment interaction in schizophrenia susceptibility [2].

The viral hypothesis has evolved considerably since its inception seven decades ago [3], now supported by converging lines of epidemiological, genetic, and neurobiological evidence. Schizophrenia impacts about 1% of the global population, representing a complex interplay between genetic vulnerability and environmental insults where viral infections emerge as significant contributors [4,5]. Therefore, the substantial heritability of the disorder, estimated at 80%, is now understood to interact critically with environmental factors [5]. As a result, the neurodevelopmental origins of the disorder are increasingly understood through the lens of immune dysregulation, and early-life viral exposures interact with the genetic risk of disrupting normal brain maturation processes [6,7].

In addition, prenatal exposure to infections and environmental stressors has been shown to raise the risk of schizophrenia, typically manifesting after puberty, a delay that could be explained by stress-induced impairments in thymic development and post-pubertal immune function. While not all individuals exposed develop the disorder, those with schizophrenia exhibit heightened susceptibility to infections and immune-mediated conditions, suggesting immunogenetic vulnerability [8].

On the other hand, prenatal maternal inflammation, characterized by elevated levels of C-reactive protein (CRP) and interleukin-8 (IL-8), similarly augments the child’s probability of schizophrenia, while anti-inflammatory cytokines including interleukin-4 (IL-4) and interleukin-5 (IL-5) exert protective effects. Experimental models demonstrate that maternal immune activation alters foetal dopamine metabolism and induces schizophrenia-like behaviours, mirroring clinical observations [9].

Emerging evidence indicates that viral infections, including severe acute respiratory syndrome coronavirus 2 (SARS-CoV-2), may influence schizophrenia development by inducing neuroinflammation that disrupts critical neurotransmitter systems. These infections perturb dopaminergic function, causing mesolimbic hyperactivity (linked to hallucinations) and prefrontal cortical deficiency (associated with cognitive dysfunction). Concurrently, inflammation impairs glutamatergic transmission via anti-N-methyl-D-aspartate (NMDA) receptor hypofunction, contributing to negative symptoms and cognitive deficits. The inflammatory cascade elevates cytokines such as interleukin-6 (IL-6) and tumour necrosis factor-alpha (TNF-α), activating the kynurenine pathway. This metabolic shift increases quinolinic acid (an excitotoxic NMDA receptor agonist) while decreasing serotonin, further destabilizing the dopamine–glutamate balance. As a result, this self-perpetuating disruption exacerbates psychotic symptoms, creating a pathological feedback loop in schizophrenia [9].

Meanwhile, some epidemiological studies further associate prenatal exposure to influenza, measles, rubella, varicella-zoster virus, and *Toxoplasma gondii* with an increased risk of schizophrenia [10].

Epidemiological patterns reveal compelling links between viruses and the likelihood of schizophrenia. Seasonal birth studies show a 5–8% growth in incidence in people who are born during the winter season and in the spring season, when viral infections peak [11], while the paradoxically higher prevalence in industrialised nations suggests complex interactions between infection control measures, vitamin D status, and pathogen exposure patterns [8]. These observations align well with the “multi-hit” hypothesis of schizophrenia, which proposes that polygenic susceptibility interacts with environmental insults in critical developmental windows to shape the risk of disease [12,13].

Among infectious agents, neurotropic viruses, particularly herpesviruses such as herpes simplex viruses (HSVs), cytomegalovirus (CMV), and human herpesvirus 6 (HHV-6), have emerged as prime candidates in the pathogenesis of schizophrenia. These viruses employ sophisticated strategies to establish persistent central nervous system infections, with herpesviruses detected in 22–43% of healthy brain tissue samples [14]. Their potential pathogenic mechanisms are diverse, ranging from direct neuronal invasion and persistence in limbic structures to molecular mimicry that triggers autoimmunity [15,16,17]. Furthermore, the resulting pro-inflammatory cytokine discharge, including interleukin-1β (IL-1β), IL-6, and TNF-α [18], and the disruption of the blood–brain barrier [19,20] can collectively lead to the neurodevelopmental abnormalities characteristic of schizophrenia.

In addition, clinical evidence supporting these mechanisms includes the identification of viral DNA in postmortem examination of brain tissue in those with psychotic diseases. For example, genomic DNA of human cytomegalovirus (HCMV) was identified post mortem in the temporal cortex of a young individual diagnosed with schizophrenia using Southern blot hybridization analysis [14,16,17]. On the other hand, epidemiological studies have demonstrated a five-fold increased risk of adult-onset schizophrenia after childhood central nervous system infections [21].

Animal models of maternal immune activation provide compelling evidence for viral contributions to schizophrenia-like phenotypes. The spiny mouse (*Acomys cahirinus*), with its precocial neurodevelopment, demonstrates how exposure to polyriboinosinic-polyribocytidylic acid during mid-gestation induces lasting cognitive deficits and microglial activation that closely mirror human schizophrenia pathology. These effects highlight that the maternal cytokine response itself, rather than specific pathogen characteristics, may be the primary driver of foetal neurodevelopmental disturbances [22].

Furthermore, experimental studies using viral mimetics like polyriboinosinic-polyribocytidylic acid have provided mechanistic insight into how immune activation during critical developmental periods may contribute to schizophrenia-related pathology. Therefore, the administration of polyriboinosinic-polyribocytidylic acid in animal models induces the upregulation of interferon-induced gene expression within the dentate region of the hippocampus, including interferon-induced protein with tetratricopeptide repeats 2 (*Ifit2*), protein kinase R (*Prkr*), MX dynamin-like GTPase 2 (*Mx2*), and interferon regulatory factor 7 (*Irf7*), while producing behavioural impairments in sensorimotor filtering and novel object identification that parallel the core features of schizophrenia. Importantly, these effects appear specific to certain cognitive domains while avoiding others, such as spatial learning and basic social interaction. Consequently, the persistence of interferon-related molecular changes under experimental conditions suggests that elevated hippocampal interferon signalling may represent a common pathway through which various environmental risk factors contribute to neuropsychiatric vulnerability [23].

Genetic studies further strengthen the viral hypothesis, particularly through immune-related loci in the major histocompatibility complex (MHC) region located on chromosome 6p21-22 [24,25]. This genomic region contains variants that influence both infection susceptibility and schizophrenia risk, including the *TNF-α -G308A* polymorphism associated with neuroanatomical changes and earlier disease onset [15,26]. The prevalent comorbidity of schizophrenia with autoimmune disorders, including celiac disease and thyrotoxicosis, and the detection of neural autoantibodies such as anti-NMDA receptor antibodies suggest overlapping pathways where viral infections may break immune tolerance [8,27,28].

On the contrary, risk loci such as catenin alpha 3 (*CTNNA3*) and zinc finger E-box binding homeobox 1 (*ZEB1*) interact with prenatal infections (e.g., CMV) to disrupt neurodevelopment [29], while others modulate dopamine receptors, glutamate transmission, and the integrity of the blood–brain barrier [30]. Blood–brain barrier dysfunction, evidenced by elevated cerebrospinal fluid (CSF) albumin quotients and kynurenic acid, may allow for a neurotoxic influx of cytokines and pathogens, exacerbating inhibition of the NMDA receptor [19,20]. In addition, antipsychotics partially mitigate this cascade by suppressing inflammatory mediators such as IL-1β and interferon-gamma (IFN-γ) and microglial activation [31,32], with adjunctive anti-inflammatory drugs (tocilizumab, minocycline) showing promise for refractory symptoms [9,33].

Moreover, clinical observations support the “mild encephalitis” model, which proposes that chronic low-grade neuroinflammation underlies up to 40% of schizophrenia cases [34]. This is evidenced by microglial activation in positron emission tomography imaging, elevated CSF cytokines (IL-6, IL-8), and altered CD4^+^/CD8^+^ ratios in peripheral blood [19]. These findings have important therapeutic implications, as antipsychotics demonstrate unexpected immunomodulatory properties by suppressing pro-inflammatory cytokines while augmenting anti-inflammatory markers such as soluble tumour necrosis factor receptor 2 (sTNF-R2) [19,31]. Furthermore, adjunctive anti-inflammatory strategies using nonsteroidal anti-inflammatory drugs and cyclooxygenase-2 inhibitors show particular promise for treatment-resistant symptoms [33,35]. Therefore, some studies have observed the benefits of anti-inflammatory agents, including nonsteroidal anti-inflammatory drugs, for positive and negative symptoms [35], and the potential utility of targeted immunomodulatory approaches in specific patient subgroups [33,36].

While nonsteroidal anti-inflammatory drugs show promise as adjunctive therapy for schizophrenia, several important limitations warrant consideration. These medications carry well-established risks—particularly gastrointestinal irritation, ulcer formation, and bleeding—that become increasingly problematic with prolonged use. Cyclooxygenase-2 inhibitors like celecoxib present additional cardiovascular concerns, while aspirin’s antiplatelet effects may exacerbate bleeding tendencies. However, the short trial durations (≤3 months) and limited sample sizes may prevent comprehensive assessment of these risks, particularly concerning potential renal or hepatic impairment. Regarding therapeutic efficacy, research demonstrates modest symptom reduction, though treatment responses vary significantly. Emerging evidence suggests enhanced effectiveness in early-stage patients and potentially greater benefits for those with elevated inflammatory markers, indicating that the response to nonsteroidal anti-inflammatory drugs may differ across patient subgroups [35].

Important questions remain regarding the temporal relationship between infectious exposures, immune activation, and symptom onset, as well as the mechanisms by which various pathogens might converge on common neurodevelopmental pathways [20]. Integration of large-scale epidemiological studies with advanced neuroimmunological techniques and longitudinal clinical assessments will be critical to elucidate these relationships and develop more effective personalised intervention strategies [33]. Furthermore, current evidence suggests that preventive approaches targeting maternal and childhood infections [11], as well as interventions to modulate immune function during critical developmental windows [33], may represent promising avenues to reduce the incidence of schizophrenia and improve long-term outcomes.

Therefore, future research that employs longitudinal designs that integrate advanced neuroimaging, viral serology, and immunogenetics will be essential to clarify these relationships. However, current evidence firmly establishes viruses as significant environmental contributors to schizophrenia—not as sole causes, but as important elements in a “multi-hit” cascade where genetic risk, neurodevelopmental timing, and immune responses converge to shape the disease trajectory. This understanding opens new avenues for personalised prevention and treatment approaches based on individual infection histories and immune profiles, offering hope for more effective interventions targeting the neuroimmune aspects of this complex disorder [12,13,20,35].

**Figure 1:**
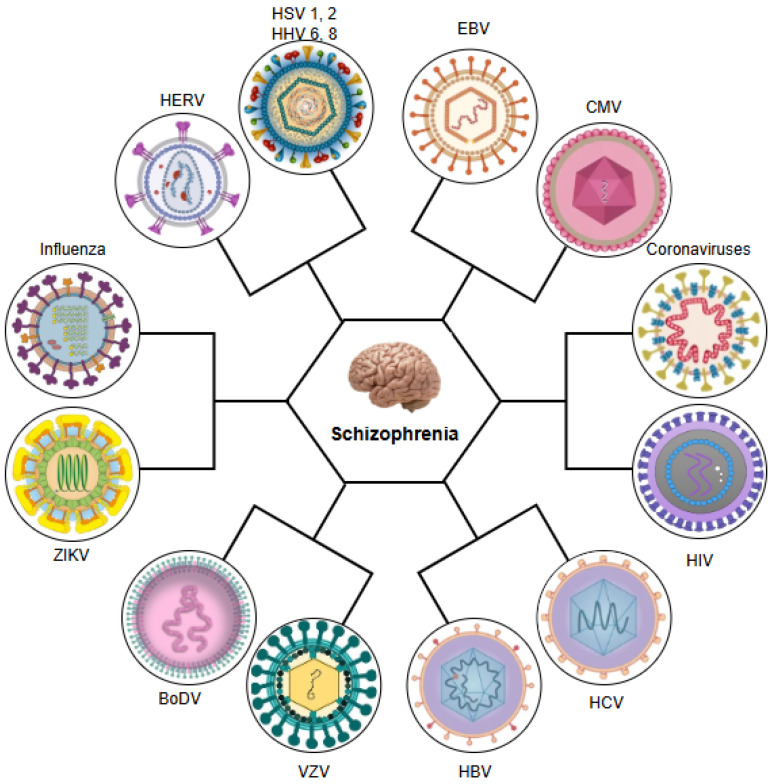
Neurotropic and systemic viruses implicated in the aetiopathogenesis of schizophrenia. HSV 1, 2: herpes simplex virus types 1 and 2; HHV 6, 8: human herpesviruses 6 and 8; EBV: Epstein–Barr virus; CMV: cytomegalovirus; HIV: human immunodeficiency virus; HCV: hepatitis C virus; HBV: hepatitis B virus; VZV: varicella-zoster virus; BoDV: Borna disease virus; ZIKV: Zika virus; HERV: human endogenous retrovirus.

Having established epidemiological evidence linking viral infections with schizophrenia, this review will now systematically examine the specific roles of key neurotropic viruses in the pathogenesis of schizophrenia. Thus, we will analyse the mechanistic pathways through which influenza virus, members of the herpesvirus family, including herpes simplex virus types 1 and 2 (HSV-1 and HSV-2), varicella-zoster virus (VZV), Epstein–Barr virus (EBV), human herpesvirus 8 (HHV-8), HHV-6, and CMV, and other viral pathogens including hepatitis B and C viruses (HBV, HCV), human endogenous retroviruses (HERVs), human immunodeficiency virus (HIV), parvovirus B19, enteroviruses, paramyxoviruses, and poliovirus, along with emerging pathogens such as Zika virus (ZIKV), Borna disease virus (BoDV), and coronaviruses, in particular SARS-CoV-2, are able to contribute to the development of schizophrenia (see Figure 1).

## 2. Herpes Simplex Virus Types 1 and 2 (HSV-1 and HSV-2)

The potential involvement of HSV-1 and HSV-2 in the pathophysiological mechanisms underlying schizophrenia has been extensively studied through epidemiological, neuroimaging, genetic, and immunological studies, revealing complex interactions between viral exposure and neurodevelopmental processes [37,38,39,40,41]. Although the evidence presents some inconsistencies [38,42,43], emerging data support the hypothesis that these neurotropic viruses may influence the development and clinical course of schizophrenia through multiple interconnected pathways [37,40,41,44].

Patients with schizophrenia show higher levels of anti-HSV-1 and 2 IgG antibodies compared to controls [45], suggesting increased exposure or impaired viral control. The virus can contribute particularly to negative symptoms and cognitive deficits [46], possibly through hippocampal dysfunction [47]. However, other investigations have shown that HSV-1-positive patients typically present with more severe illness characteristics, including an extended disease course, heightened positive symptomatology, and lower quality of life, suggesting that viral infection could identify a distinct clinical subgroup [48].

Epidemiological studies examining maternal HSV-2 infection have yielded mixed findings on the risk of schizophrenia in offspring [38,42,49]. The virus’s different route of transmission (primarily sexual) and reactivation pattern (from lumbosacral ganglia) may account for its more restricted neuropsychiatric impact compared to HSV-1 [50]. Thus, some investigations report associations potentially mediated by immune dysregulation throughout essential phases of foetal brain maturation [49,51], with mothers of individuals who develop schizophrenia showing increased concentrations of IgG and IgM immunoglobulins indicative of persistent HSV-2 infection [51]. However, these results have not been uniformly reproduced across all investigations [38,52], with several large cohort analyses demonstrating that initial associations become nonsignificant after controlling for confounding factors such as parental psychiatric history [42,49], suggesting that prenatal exposure to HSV-2 may not represent a major aetiological factor in schizophrenia. These studies employed logistic regression and cohort analyses to assess the relationship between maternal IgG and IgM antibodies and schizophrenia risk. Buka et al. [51] reported significant associations between IgG and IgM antibodies against HSV-2 and schizophrenia risk using regression models adjusted for demographic factors. In contrast, other studies [38,42,49] also examined maternal HSV-2 antibodies but applied more extensive covariate adjustment in multivariate models, which led to attenuated effects. The discrepancies in findings may stem from differences in antibody detection methods, sample sizes, or covariate adjustment strategies [38,42,49,51].

On the other hand, HSV-2 exposure combined with other infections such as *C. pneumoniae* leads to accelerated cortical thinning and a reduction in hippocampal volume [53]. In particular, these structural changes may represent a neuroanatomical substrate for the cognitive decline observed in individuals exposed to HSV, particularly those who develop schizophrenia [53,54].

However, the possible involvement of HSV-2 in increasing susceptibility to schizophrenia appears to be mediated by gene–environment interactions involving NMDA receptor polymorphisms. Several investigations have presented strong evidence indicating that genetic variants in glutamate ionotropic receptor NMDA type subunit 2B (*GRIN2B*), which encodes the NR2B subunit of NMDA receptors, may modify susceptibility to schizophrenia in offspring of HSV-2-seropositive mothers. The analysis of three Danish case–control cohorts revealed significant interactions between maternal HSV-2 seropositivity and several single-nucleotide polymorphisms of *GRIN2B*, particularly within the 3’ region of the gene. These results indicate that the maternal immune response to HSV-2 reactivation during pregnancy, rather than direct viral transmission, may disrupt foetal neurodevelopment through effects on the function of the NMDA receptor. This is particularly plausible given the significant involvement of NR2B-expressing receptors in the initial stages of brain maturation and synaptic plasticity [40].

In contrast to HSV-2, HSV-1 appears to be more consistently linked to schizophrenia through distinct neurobiological mechanisms (see Table 1 and Table 2) which influence the pathogenesis of the disorder through more direct neuroinflammatory and neurodegenerative mechanisms [37,44,55]. Therefore, the virus can exert these effects through multiple pathways: direct neuronal damage during reactivation, chronic neuroinflammation, and disruption of neurotransmitter systems, including dopamine and glutamate [37,41]. Furthermore, it has been shown that HSV-1 can impair sensorimotor gating in early postnatal infection models, mirroring the deficits seen in schizophrenia [56,57]. Moreover, viral reactivation in the brain can lead to elevated levels of monoamines (dopamine, norepinephrine, and serotonin), which are implicated in psychotic symptoms [37]. On the other hand, animal-based research further indicates that HSV-1 in its latent state can result in progressive neuronal injury even without detectable viral replication. Studies in BALB/c mice revealed that persistent HSV-1 latency in the trigeminal ganglia has been linked to reductions in neuronal size, reduced density, and chronic inflammation, suggesting that persistent viral presence may contribute to neurodegenerative changes [55]. The virus demonstrates particular tropism for the hippocampus and limbic system, brain regions consistently implicated in schizophrenia pathology [47,58,59], entering neurones through nectin-1 receptors that exhibit specific developmental expression patterns [59]. This regional vulnerability may explain the ability of HSV-1 to disrupt neurodevelopmental processes through several potential pathways, including direct viral effects on neural progenitor cells, where latent infection may interfere with differentiation programmes through epigenetic modifications involving the viral thymidine kinase gene [60]. Furthermore, HSV-1 infection disrupts nectin-1-mediated cell adhesion, which may contribute to the aberrant neural connectivity observed in schizophrenia [59]. Neurotropism, defined as the virus’s capacity to selectively infect and persist within neural tissues, including specific brain regions, may underlie such neuropathological effects. Therefore, HSV-1 neurotropism for the frontal and temporal cortices positions it to disrupt the neural circuits critical for working memory and executive function. Potential mechanisms include protein aggregation, autophagy dysregulation, oxidative stress, mitochondrial dysfunction, and apoptosis [61,62,63,64].

In contrast, interleukin-18 (IL-18) has emerged as a putative mediator of the impact of HSV-1 in the context of schizophrenia. Elevated IL-18 concentrations have been documented in individuals with schizophrenia, and genetic variations in the components of the IL-18 signalling pathway appear to interact with the serostatus of HSV-1 to influence the risk of disease [74]. This suggests that HSV-1 infection may amplify neuroinflammatory processes in genetically susceptible individuals, potentially causing the observed neuroanatomical and functional impairments [41,74]. Moreover, the inflammatory hypothesis of schizophrenia gains support from studies that demonstrate synergistic effects of HSV-1 seropositivity and increased CRP levels regarding the degree of cognitive dysfunction observed in patients with schizophrenia. Thus, patients exhibiting both risk factors show 2.35 times increased odds of significant cognitive dysfunction in comparison with seronegative individuals exhibiting normal CRP concentrations [98].

Neuroimaging studies provide compelling evidence for HSV-1’s neuropathological effects [37,44,72,73]. Thus, patients with schizophrenia and elevated HSV-1 antibody titres have been shown to exhibit greater cortical atrophy, reduced frontal lobe volumes, and morphological abnormalities in the corpus callosum compared to seronegative patients [73]. Furthermore, structural grey matter deficits have been shown within the dorsolateral prefrontal cortex and the anterior cingulate gyrus regions among seropositive patients with schizophrenia compared to their seronegative counterparts [37].

In contrast, individuals at an ultrahigh risk of psychosis with HSV-1 exposure exhibit volumetric decreases in the cuneus compared to nonexposed ultrahigh-risk individuals and healthy controls. These cuneal abnormalities may be related to visual processing deficits and abnormalities in eye movement observed in schizophrenia, which may reflect the broader self-monitoring impairments characteristic of psychotic disorders [105]. In particular, the reduced volume of grey matter in the anterior cingulate and cerebellar regions that has been observed among HSV-1-positive individuals resembles to that observed in post-encephalic patients, suggesting potential shared neuropathological mechanisms [72,106]. These structural alterations are correlated with measurable cognitive impairments in multiple domains, including working memory, executive function, and processing speed [44,72,107,108], and longitudinal investigations reveal a progressive decline in executive function and a reduction in grey matter within the posterior cingulate regions in subjects in the first episode of schizophrenia who are positive for HSV-1 [44]. In particular, similar cognitive deficits have been observed in HSV-1-seropositive healthy individuals [67,68,69,70,71], suggesting that these effects may reflect general viral neuropathology that interacts with vulnerability factors for schizophrenia rather than representing disease-specific processes [64,67].

Although these deficits manifest similarly in both patients with schizophrenia and healthy populations, greater severity has been observed in the former [64].

Furthermore, longitudinal studies in adolescents have demonstrated that HSV-1 seropositivity predicts poorer cognitive performance, particularly in immediate memory and executive function, independent of socioeconomic or lifestyle factors [71]. These findings align with reports of progressive cognitive decline and worse negative symptoms in HSV-positive cases [46], although not all studies confirm these associations [43]. Moreover, another longitudinal study has demonstrated that HSV-1-seropositive individuals showed significantly poorer performance at baseline in the domain of emotion identification and discrimination (EMOD) compared to seronegative individuals (B = −0.28, *p* = 0.018). More importantly, they exhibited a steeper decline in EMOD performance over time (B = −0.15, *p* = 0.033), indicating a progressive worsening in this domain of social cognition. Complementing these findings, a randomized controlled trial evaluating valacyclovir treatment in HSV-1-infected patients with schizophrenia demonstrated a significant improvement in EMOD scores among the treatment group compared to placebo (Cohen’s d = 0.43, *p* = 0.048). These results underscore the longitudinal impact of HSV-1 infection on social cognitive functioning and suggest the potential reversibility of EMOD deficits through targeted antiviral intervention [109]. Therefore, these findings hold clinical significance given the established relationship between EMOD and functional outcomes in schizophrenia [110,111].

The neurobiological mechanisms underlying the cognitive effects of HSV-1 likely involve both direct viral pathology and immune-mediated processes. During latency, HSV-1 establishes persistent infection in the sensory ganglia with potential retrograde transport to the frontotemporal regions through olfactory pathways [112]. Periodic reactivation may cause cumulative neuronal damage through several pathways: repeated subclinical viral replication in circumscribed brain regions, neurodevelopmental alterations from early-life exposure, or systemic cytokine release that triggers neuroinflammation [44,63,84,103]. Furthermore, postmortem studies have demonstrated the existence of HSV-1 DNA in neural tissue from individuals without a history of encephalitis [113], while rodent models demonstrate latent central nervous system infection [104], supporting these mechanistic possibilities.

The immunological consequences of HSV infection represent another critical pathway to schizophrenia pathology [41,74,75]. Viral recognition by microglial Toll-like receptors (TLR2, TLR3, TLR9) triggers robust neuroinflammatory responses characterised by elevated key pro-inflammatory mediators such as IL-6, TNF-α, and IL-1β [75], patterns consistently observed in patients with schizophrenia. This persistent low-grade neuroinflammation manifests itself as widespread microglial activation in first-episode psychosis cases, correlates with cognitive deficits in HSV-seropositive individuals [73,75], and may be modulated by certain antipsychotic medications, such as risperidone and haloperidol, which demonstrate cytokine-suppressing effects [75]. Maternal immune activation models reveal that prenatal contact with viral mimics such as polyriboinosinic-polyribocytidylic acid induces schizophrenia-like behavioural alterations observed in the progeny, such as sensorimotor gating deficits and cognitive impairments that parallel the core characteristics of the disorder, supporting the plausibility of immune-mediated developmental disruption [46,60].

Genetic studies further strengthen the HS–-schizophrenia connection [39,40,78], revealing overlapping risk loci in the major histocompatibility complex that influence both infection susceptibility and disease vulnerability [54,60,77]. Whole-exome sequencing has identified multiple genes associated with HSV infection pathways in cases of familial schizophrenia [78], while elevated expression of Fyn kinase (Fyn), a known HSV binding partner, in the patient’s prefrontal cortex suggests specific molecular interactions [78,102]. Furthermore, upregulation of Fyn in infected placental tissue further supports its role in viral pathogenesis and neurodevelopmental disruption [102]. These genetic insights help explain why only a subset of individuals exposed to HSV develop psychotic disorders and highlight potential gene–environment interactions that can determine neurodevelopmental outcomes [40,76].

Furthermore, genetic susceptibility studies reveal a significant overlap between schizophrenia-associated genes and HSV-1 interactome components, particularly in immune and inflammatory pathways. The HSV-1 interactome shows specific enrichment in genome-wide association studies of schizophrenia, with particular viral entry receptors such as the neuropilin-1 (NRP1) receptor appearing in schizophrenia datasets. This suggests that host genetic factors may mediate viral effects on neurodevelopment [39].

However, other immunogenetic studies reveal that specific HLA alleles associated with schizophrenia protection show stronger binding affinities to HSV-1 antigens, suggesting that impaired viral clearance may contribute to disease risk [77]. This is further supported by electron microscopy evidence of HSV-1 particles in foetal neuronal nuclei from pregnancies of women with schizophrenia, indicating potential prenatal neurodevelopmental disruption [93].

Genetic studies further reveal potential moderators of the neuropathological impact of HSV-1. The major histocompatibility complex class I polypeptide-related sequence B (*MICB*) *rs1051788* polymorphism demonstrates pleiotropic effects, with allele A correlated with an elevated likelihood of schizophrenia and allele G in relation to elevated antibody titres for HSV-1 in healthy subjects (*p* = 0.006, OR = 2.7). Therefore, the A allele may impair natural killer (NK) cell-mediated viral control via an aspartic acid-to-asparagine substitution in *MICB’s* NKG2D binding domain, potentially exacerbating neuroinflammation, while the G allele correlates with robust antibody responses, suggesting allele-specific immune modulation. These findings imply that *rs1051788* influences both schizophrenia susceptibility and HSV-1 immune responses [76,114]. In particular, the combined role of HSV-1 infection and *MICB* genotype in prefrontal grey matter reduction appears more evident in individuals diagnosed with schizophrenia relative to controls (14.9% volume loss vs. 6.4% without genetic correction), suggesting that gene–environment interactions may partially explain neuroanatomical variability in schizophrenia [76].

At the neurochemical level, HSV infection appears to converge on NMDA receptor dysfunction, a central pathway in schizophrenia pathophysiology, through multiple mechanisms, including the production of the endogenous antagonist kynurenic acid and triggering autoimmune responses against NMDA receptors after encephalitic episodes. This receptor hypofunction disrupts the cortical excitatory–inhibitory balance and may contribute to psychotic symptoms [66]. Moreover, the virus demonstrates a remarkable ability to establish latent infections in neuronal tissues, as demonstrated in human induced pluripotent stem cell-derived neurone models where it enters a quiescent state while inducing specific transcriptional changes in glutamatergic signalling pathways, potentially exacerbating NMDA receptor hypofunction. Consequently, these molecular alterations may be the basis for the well-documented correlation between contact with herpes simplex viruses and mental functions, particularly the working memory deficits characteristic of schizophrenia [66,88]. Additionally, the neuroinflammatory consequences of infection further compound these effects, with imaging studies demonstrating persistent microglial activation in the hippocampal regions during acute psychosis [96].

The therapeutic implications emerging from these findings remain preliminary but potentially significant [41,48,63]. Although antiviral treatment has shown promise in reducing the risk of dementia among individuals with symptomatic herpes infections, comparable evidence in schizophrenia is lacking [53]. Therefore, valacyclovir antiviral treatment has shown modest cognitive benefits in some clinical trials [48,63]. Therefore, outcomes have differed in various research efforts, possibly reflecting the challenges of targeting latent viral reservoirs or heterogeneity in patient populations [41,48]. Interestingly, a randomized study with blinded participants and researchers adopting a placebo-based design using elevated doses of valacyclovir (8 g/day) in patients with schizophrenia and active psychosis found significant reductions in microglial activation, as measured by positron emission tomography of translocator protein (TSPO), particularly within the amygdala, hippocampal, and cingulate regions of the brain. However, despite these neuroinflammatory changes, no symptomatic or cognitive improvements were observed, possibly due to the short duration of treatment or insufficient penetration of central nervous system drugs at lower doses [41]. Therefore, antiviral therapy can represent a new treatment approach for cognitive deficits in a subset of patients [48].

Alternative approaches, including immunomodulation strategies and combination therapies targeting both viral and inflammatory components, may hold promise [41], particularly for individuals with evidence of active immune dysregulation [41,108]. Therefore, epigenetic modulators such as lysine-specific histone demethylase 1 inhibitors (e.g., tranylcypromine) can suppress HSV thymidine kinase reactivation [60], while microglial-targeting compounds (corilagin, olomoucine) could mitigate virus-induced neuroinflammation [75]. Furthermore, strategies targeting NMDA receptor dysfunction, either through glycine site modulation or kynurenine pathway intervention, may be particularly relevant for patients with evidence of HSV exposure [66]. Therefore, future research should focus on identifying which patient subgroups could benefit the most from these interventions, potentially using genetic markers associated with NMDA receptor activity or immunological signalling routes [40,41].

In conclusion, current evidence positions HSV-1 as a plausible environmental contributor to the pathogenesis of schizophrenia [37,40,41,44], particularly when it interacts with genetic vulnerability factors during critical neurodevelopmental periods [40,76]. Although not all HSV-1-exposed individuals develop schizophrenia and not all studies find consistent associations [38,42,43], the convergence of epidemiological, neurobiological, and genetic findings suggests that these viruses may represent one component of the multifactorial aetiology of the disease. Future investigations should aim to elucidate the temporal relationships between infection, immune response, and symptom onset [41], identify reliable viral contribution biomarkers [76], and develop targeted interventions for at-risk individuals based on personalised risk profiles incorporating genetic and environmental factors [40,41]. Additionally, the potential interaction between HSV-1 and additional contributing factors, including genetic vulnerability and substance use, warrants investigation [72]. Furthermore, the potential synergy between HSV-1 and other pathogens including *Toxoplasma gondii* or inflammatory states, warrants investigation, given their shared pathways to neuronal dysfunction [115,116]. Meanwhile, the development of vaccines to prevent primary HSV-1 infection may represent a more promising preventive approach [108].

## 3. Varicella-Zoster Virus (VZV)

VZV is the third human alpha-herpesvirus, as well as the pathogen that causes chickenpox upon first exposure and shingles (herpes zoster) when reactivated [117,118]. After the initial illness subsides, VZV remains dormant within the spinal ganglia [118]. Once the virus enters a latent state, keeping it suppressed requires constant involvement from surrounding virus-specific CD8^+^ T cells and glial cells, which inhibit lytic gene expression by releasing granzyme B and IFN-γ. A latency-associated transcript (*LAT*) is also expressed, along with several microRNAs (miRNAs), which are key features of alpha-herpesvirus latency. Even though the exact function of *LAT* transcripts remains unclear, research suggests that such non-coding RNAs play a role in preventing programmed cell death within neurons harbouring the infection and regulating the virus’s ability to reactivate [117]. In terms of reactivation, it can be triggered by various stressors, such as metabolic, hormonal, and environmental changes [119]. Interestingly, VZV produces six various transcripts and proteins during latency. Among them, open reading frame 63 (ORF63) is one of the most plentiful proteins, remaining mostly in the cytoplasm during latency but shifting predominantly to the nucleus during lytic infection [117].

More recently, polymerase chain reaction techniques have been used on spinal ganglion tissues to determine whether latent VZV resides in neurones, satellite cells, or both. It has been demonstrated that the virus is primarily, if not exclusively, found in neurones, with approximately two to five viral copies per infected neurone [120]. Additionally, recent studies suggest a link between VZV infection and the development of NMDA receptor-specific antibodies, which have been associated not only with post-viral encephalitis, but also with epilepsy [121,122].

VZV infection may also be associated with depression and anxiety [123,124]. Studies have shown that VZV antibodies are considerably more common in individuals with psychotic depression [125]. However, findings on the role of this virus in the onset of depression remain inconsistent. For instance, Pang et al. [126] assessed the relationship between diagnostic assessments of VZV infection, including shingles, among pregnant women at any point during gestation and the likelihood of depression in the children. Their study did not detect a correlation between maternal herpesvirus or shingles and depression in offspring [126]. Similarly, other studies utilizing viral DNA detection in cerebral tissue was unable to identify any correlation between VZV exposure and depression [127].

The association between maternal herpesvirus exposure and offspring depression demonstrates significant methodological inconsistencies across studies. While serological approaches frequently identify associations between maternal HSV and VZV antibody levels and child depression risk, clinical diagnostic studies of prenatal infections typically report null findings. Similarly, tissue-based analyses fail to demonstrate links between persistent central nervous system viral presence (CMV or VZV) and depression. These conflicting results may stem from differences in exposure assessment techniques, target virus selection, and case ascertainment methods in offspring. The variation in findings highlights the need for standardized, prospective designs that integrate multiple exposure measures with rigorous neuropsychiatric phenotyping [123,124,125,126,127].

On the other hand, the link between VZV and schizophrenia has been extensively investigated, with the findings remaining inconsistent. Divergent findings may arise from differences in the timing of exposure during gestation, as immune responses and neurodevelopmental susceptibility vary across developmental stages. Moreover, variability in maternal and foetal immune reactivity, as well as limited standardisation across animal models, can contribute to inconsistent outcomes. Furthermore, methodological limitations—such as the use of ecological study designs, where infection exposure is inferred at the population level without individual confirmation—reduce the strength of causal inference and may also explain conflicting results [128,129]. Evidence indicates that perinatal contact with VZV significantly heightens the likelihood of schizophrenia onset [128]. For instance, research carried out by Torrey et al. demonstrated a notable association between the emergence of schizophrenia in children and maternal varicella-zoster infection during the fifth to seventh months of gestation [129].

Conversely, numerous studies have reported negative findings regarding the association between schizophrenia and VZV. Several of these investigations have assessed VZV infection through serological methods [125,130,131,132,133,134]. Furthermore, Fukuda et al. [134] did not observe notable changes in antibody concentrations against VZV between the acute psychotic phase and after an eight-week therapeutic intervention in individuals diagnosed with schizophrenia. Additionally, a prospective investigation that examined the impact of IgG antibodies against VZV on progression to psychosis during a follow-up interval of 6.46 years identified no correlation between the Herpesviridae antibody profile and the risk of transition. However, the study demonstrated that *Toxoplasma gondii* infection was associated with a 3.6-fold elevation in the likelihood of transition [135].

**Figure 2:**
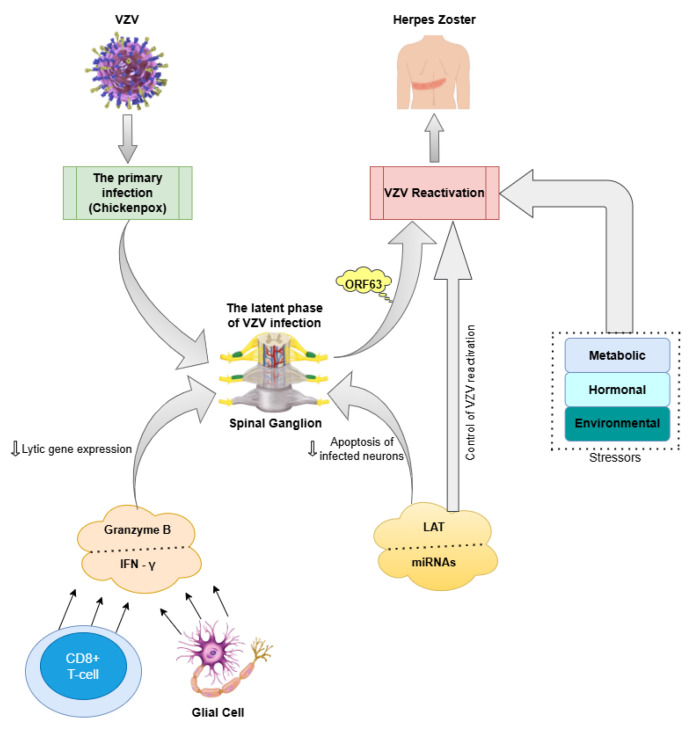
Hypothesized pathogenic pathways linking VZV infection to schizophrenia. VZV: varicella-zoster virus. IFN-γ: interferon-gamma. LAT: latency-associated transcript. MiRNAs: microRNAs. ORF63: open reading frame 63; (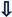): inhibition.

Other studies investigating the presence of viral genetic material in postmortem brain samples from individuals with schizophrenia [136,137,138,139] have not found a connection between VZV infection and the condition, while several studies have detected VZV in human brain tissue [101]. These hypothesized mechanisms which reveal the association between VZV and schizophrenia can be seen in Figure 2.

## 4. Epstein–Barr Virus (EBV)

EBV, also referred to as human herpesvirus 4 (HHV-4), is part of the Herpesviridae family, characterized by a great prevalence throughout the global population, with evidence reporting that over 90% of individuals of adult age worldwide are seropositive for this infection [140,141,142]. First-time exposure to EBV is typically characterized by a self-limiting febrile illness accompanied by lymphadenopathy, a clinical presentation commonly known as infectious mononucleosis. After resolution of the acute phase, EBV can persist within epithelial cells, monocytes, and host B and T lymphocytes. Transmission between individuals occurs primarily through salivary viral shedding [143].

The virus can establish a latent state at various anatomical sites, including the central nervous system, where reactivation has been related to encephalitis and the triggering of brain-specific immune responses [144,145]. Reactivation of EBV can happen during episodes of physical or psychological stress and is typically characterized by a marked increase in virus-specific antibody titres, despite the lack of virologically detectable presence [146].

EBV infections have been implicated in the pathogenesis of various autoimmune conditions, such as multiple sclerosis, fibromyalgia, systemic lupus erythematosus, and autoimmune encephalitis [147,148]. In numerous instances of autoimmune conditions, the immunological response to EBV in affected individuals exhibits atypical characteristics, diverging from the response observed in healthy controls [149,150].

On the other hand, a significant proportion of people with EBV-related diseases experience psychiatric manifestations throughout the progression of their condition. For example, in systemic lupus, psychosis affects over 20% of patients, while cognitive dysfunction has been documented in over 80% of cases. In addition, mood disturbances, delirium, anxiety, seizures, and cerebrovascular disease have also been reported [151]. Similarly, cognitive impairment and psychosis have been observed to be highly prevalent among individuals with multiple sclerosis [152,153,154].

EBV infection has also been associated with the onset of depressive symptoms [124], although some studies have reported negative results [155,156]. Specifically, evidence suggests that reactivation of EBV is correlated with an elevated occurrence of depression among mothers in the initial and later phases of gestation [157,158], while salivary EBV DNA shedding in adolescent females is likewise linked to depressive symptomatology [159]. On the contrary, a recent paediatric study found no meaningful relationship between EBV seropositivity or antibody titres and the presence of depressive disorder [155]. Similarly, a long-term observational study with an 11-year follow-up found no substantial link to earlier contact with EBV, along with CMV, HSV-1, and *Toxoplasma gondii*, and the subsequent development of depressive disorders [156].

Evidence on the impact of EBV infection on schizophrenia remains inconclusive. EBV has been extensively investigated over the years as a potential aetiological factor in this psychiatric condition, particularly in light of the elevated seroprevalence reported among individuals diagnosed with schizophrenia [160,161]. On the contrary, other studies have failed to detect the detection of EBV in postmortem frozen brain tissue specimens from individuals with schizophrenia [137].

The likelihood of an individual developing the disorder may also be influenced by a genetic predisposition to manifest neuropsychiatric effects in response to infection [91]. Thus, genetic investigations have demonstrated that the combined presence of elevated levels of antibodies targeting EBV virions and a genetic predisposition to schizophrenia is linked with an over 8.5-fold rise in the likelihood of developing the disorder [79]. Moreover, it has been proposed that a portion of the genetic susceptibility to psychosis may be attributable to an underlying genetic predisposition to infections [162,163].

Another study that supported the hypothesis of immune dysfunction and EBV infection as a cause of psychosis has been conducted by Khandaker et al., who have demonstrated that early childhood contact with EBV may result in a heightened likelihood of developing definitive psychotic experiences during adolescence [83]. These results align with a further important investigation that involved a considerable prospective cohort of 1176 adolescents and similarly demonstrated a link between the presence of EBV, as confirmed by serological analysis, and positive psychotic symptoms, but only in men [91]. However, longitudinal research has shown that individuals who report psychotic experiences during childhood may face up to a 16-fold increased likelihood of developing psychotic disorders in adulthood [164,165]. Moreover, a link was observed between antibody levels targeting EBV virion components and tobacco use in people with schizophrenia [79]. Similar interactions have been reported in other EBV-related diseases, like multiple sclerosis, likely due to the immunomodulatory effects of smoking [166].

The pathways through which EBV exposure may contribute to an increased susceptibility to psychosis remain poorly understood (see Table 2) [91]. These hypotheses may be explained by the fact that early-life EBV infection could have detrimental effects on neuronal survival and function by priming microglia to exhibit a dysregulated response to future infections [83]. On the other hand, EBV infection, similar to HSV infection, may induce the transactivation of endogenous retroviruses, which may play a role in the modulation of host gene expression [87].

Conversely, infectious agents can exert their effects indirectly by triggering systemic cytokine responses and stress-related mechanisms, whose levels have been shown to exhibit altered expression patterns in individuals diagnosed with schizophrenia [91]. Moreover, these pro-inflammatory signalling molecules in the brain can contribute to triggering the innate immune system [167,168]. It is also plausible that immune-related genes influence the nature of the post-infection inflammatory process, which subsequently leads to disruptions in cerebral development and function [83].

In addition, increased concentrations of inflammatory biomarkers in the mother’s circulatory system during gestation have previously been linked with an elevated probability of psychosis in adult offspring [167,168]. Furthermore, acute episodes of psychosis have been correlated with elevated serum concentrations of pro-inflammatory signalling molecules, including IL-6, and evidence of microglial activation, which was identified by positron emission tomography images of the brain [169,170]. Preclinical studies conducted on animal models also indicate a potential role of prenatal inflammatory processes in the development of psychotic conditions [171].

However, studies indicate that EBV infection may lead to cognitive deficits, a key characteristic of schizophrenia. Thus, a current investigation has identified a potential relationship between EBV infection and the onset of cognitive impairment in individuals with schizophrenia, particularly in relation to social cognition. As a result, diminished cognitive functioning has been linked to increased concentrations of antibodies targeting not only the complete EBV virion but also the viral capsid antigen (VCA) protein and the EBV nuclear antigen-1 (EBNA-1) protein. Specifically, elevated antibody levels against the EBNA-1 protein have been associated with decreased performance in the working memory domain, while higher antibody levels against the whole EBV virion have been linked to reduced scores in the cognitive processing speed domain [172].

Conversely, further research has identified a distinct relationship pattern between antibody levels targeting specific EBV antigens and schizophrenia, indicating an impaired immune reaction to EBV infection that could contribute to the immunopathogenesis of schizophrenia and related diseases. Patients with schizophrenia exhibit an abnormal immune reaction to EBV infection, showing elevated concentrations of antibodies directed against the entire EBV virion and VCA, while no significant increase in EBNA-1 antibodies have been reported relative to control subjects. Unlike healthy individuals who develop proportional responses to both antigens, this imbalanced pattern implies either ongoing viral activity or defective latency establishment. These immunological disturbances could promote neuroinflammation, potentially explaining the observed psychiatric symptoms through mechanisms similar to other EBV-related autoimmune conditions, where immune factors interfere with normal brain function [79]. The lack of a correlation between schizophrenia and EBNA antibodies was also reported by DeWitte et al. [173]. This observation is particularly noteworthy given that EBNA-1 peptide sequences have been recognised as primary mediators of cross-reactivity with neural targets, including myelin basic protein [174] and nuclear ribonucleoprotein L [175]. Accordingly, it is conceivable that individuals exhibiting a diminished immune response to EBNA-1, along with heightened reactivity to other EBV-derived proteins, may be at elevated risk of immune-mediated pathology in the central nervous system [176].

The precise biological pathways explaining the relationship between cognitive abilities and variability in immune responses to EBV proteins remain unclear. Potential explanations for this relationship include the genetic characteristics of the viral strain, variations in the timing of primary infection, and individual differences in host immune responses to EBV, shaped by both genetic predisposition and external influences [177,178].

The negative influence of EBV infection on cognitive performance was also demonstrated by Steel et al. [179], whereas other investigations have not found a significant association [180,181,182]. Moreover, a prospective study conducted in a cohort of 1084 adolescents with an average age of 16 years found no meaningful correlation between cognitive performance and EBV infection [71].

However, seropositive status for EBV has been related to lower IQ, as well as a higher likelihood of onset of substance use disorder in children. This association was identified in a prospective cohort study involving 569 children, both male and female, whose fathers had or did not have substance use disorder. These findings suggest that neurotropic infections during childhood, such as EBV, could potentially impact not only cognitive growth, but also susceptibility to behavioural disorders, including substance use disorder [183].

Concerning the aspect of schizophrenia potentially influenced by EBV infection, research has indicated an association between elevated antibody levels against EBV virion and VCA proteins and a heightened occurrence of the deficit syndrome subtype within the disorder [79].

The precise neurobiological pathways linking elevated EBV virion antibody levels to schizophrenia remain unclear [79]. One hypothesis suggests that psychiatric manifestations may result from neuroinflammatory processes affecting the brain, encompassing disruptions within neurotransmitter receptor signalling, as observed in various autoimmune neurological disorders [92]. An alternative explanation posits that individuals with dysregulated immune responses to EBV may have experienced prior viral replication within the central nervous system. This is supported by documented cases of psychosis associated with EBV encephalitis as well as elevated concentrations of EBV VCA antibodies within the CSF of certain patients with mental health conditions [79].

Further findings reinforcing the contribution of latent EBV infection in the aetiopathogenesis of schizophrenia arise from a recent study that demonstrated intrathecal synthesis of EBV-specific antibodies in individuals with chronic schizophrenia spectrum disorders, in comparison with those newly diagnosed. The findings also indicate the presence of a multifaceted immune reaction within the central nervous system among 3% of individuals with chronic illness, in contrast to 0% among those with schizophrenia spectrum disorders at the first episode, implying the contribution of additional immunological mechanisms [184]. Moreover, schizophrenia genome-wide association studies have detected the strongest genetic signal within the MHC locus on chromosome 6, which contains the complement component 4 (*C4*) gene. In particular, the risk of schizophrenia has been partially attributed to allelic variations in *C4*, a key player in viral inactivation. This is particularly significant given that complement receptor 2, which serves as a receptor for EBV, interacts with this pathway, suggesting a plausible pathway by which EBV could play a role in the onset of schizophrenia [101].

Conversely, some studies have not reported significant differences in EBV antibody levels in serum or intrathecal fluid between unmedicated patients with recent-onset schizophrenia and healthy controls [133]. In addition, a separate analysis involving individuals with early-stage psychosis did not find a correlation with the seropositivity of EBV [185]. Furthermore, a meta-analysis synthesising data from multiple studies on pathogens in schizophrenia did not reveal greater exposure to EBV among affected individuals [130]. Another study that examined the possible involvement of EBV infection in the aetiology of postpartum psychosis found no supporting evidence, concluding that exposure to this pathogen is not involved in the pathophysiology of the disease [186]. Similarly, a longitudinal cohort study involving 96 patients classified as ultrahigh risk for psychosis evaluated the impact of previous contact with neurotropic infectious agents on the likelihood of progression to a psychotic disorder. The findings did not find a meaningful correlation between seropositivity for viruses of the Herpesviridae family and the risk of developing psychosis over time [135].

In conclusion, despite some negative findings, the potential involvement of EBV in the pathogenesis of schizophrenia, particularly in relation to cognitive deficits, warrants further investigation [71,179,180,181]. Current pharmacological interventions remain insufficient in addressing the cognitive impairments observed in individuals with schizophrenia [172]. Notably, EBV exhibits a lower sensitivity to commonly used antiviral agents such as valacyclovir and its prodrug, acyclovir, in comparison with other herpesviruses like HSV-1 and HSV-2. Consistently, previous trials have not demonstrated a cognitive benefit of valacyclovir in patients with early-stage schizophrenia [48,172]. Nonetheless, the development of more potent antiviral compounds targeting EBV offers promising therapeutic avenues. Several of these agents are currently in advanced stages of development and can contribute to the prevention or amelioration of EBV-associated cognitive dysfunctions in vulnerable populations [187]. In particular, compounds such as valproic acid and valpromide have shown the ability to inhibit EBV reactivation during the lytic phase in Burkitt lymphoma cell models. Furthermore, clozapine, a second-generation antipsychotic frequently used to treat schizophrenia resistant to standard therapies, and its metabolite, desmethylclozapine, have been shown to suppress the manifestation of lytic EBV genes (including *BMLF1*, *BZLF1*, and *BRLF1*) in a dose-dependent manner [188].

These findings highlight the need for a more thorough comprehension of the interaction between latent viral infections and neuropsychiatric conditions. Elucidating the role of EBV in the neurophysiology of schizophrenia could ultimately contribute to the emergence of innovative preventive and curative strategies [79].

## 5. Cytomegalovirus (CMV)

CMV, another neurotropic virus belonging to the Herpesviridae family, has been increasingly suggested to be involved in the pathogenesis of schizophrenia through multiple pathways, including congenital infection, immune dysregulation, and neurodevelopmental disruption (see Table 2) [80]. Taking into account the lifelong latency capacity of CMV, emerging evidence suggests that its effects on the central nervous system may contribute to psychiatric disorders, particularly schizophrenia [189,190]. Therefore, the virus exhibits a strong tropism for neural progenitor cells and limbic structures, including the hippocampus and temporal cortex, regions critically involved in schizophrenia pathology. However, evidence of CMV’s tropism for neural progenitor cells stems mainly from animal models, especially murine CMV (MCMV), which targets neural progenitor cells in the ventricular and subventricular zones, disrupting their proliferation and differentiation. In vitro studies using human and mouse neural progenitor cells support this tropism, but in vivo confirmation in humans is limited. Moreover, interspecies differences, such as MCMV’s β1 integrin-mediated entry, further limit translational relevance. Although CMV has been detected in neural progenitor cell-rich regions of infected foetal tissues, mechanistic insight is lacking, highlighting the need for human-relevant models [16,80,89,90,191,192,193]. Furthermore, intrauterine CMV infection is a well-established driver of foetal neurodevelopmental impairments, such as microcephaly and polymicrogyria, which share similarities with structural brain alterations observed in schizophrenia [81,82]. This association is supported by serological studies that demonstrate elevated CMV antibody titres in individuals with schizophrenia, particularly those with the deficit subtype characterised by primary negative symptoms [194,195,196]. However, the findings in all studies remain inconsistent, with some reporting no substantial variation in CMV seropositivity between affected individuals and healthy subjects [197], highlighting the need for additional studies to elucidate the underlying aspects of this relationship.

The potential mechanisms linking CMV with schizophrenia are multifaceted. Maternal immune activation during pregnancy, triggered by CMV infection, may disrupt foetal brain development through inflammatory cytokines and altered neurogenesis [85]. CMV’s potential to induce latent infection in neural progenitor cells and reactivate under conditions of immune compromise further supports its role in neurodevelopmental disorders [90,94]. Transgenic models have shown that the transcription of CMV early genes in the developing brain can lead to neuronal migration defects and synaptic dysfunction, paralleling the neuropathological features of schizophrenia [82]. Additionally, molecular mimicry between CMV peptides and human neurodevelopmental proteins, such as glutamic acid decarboxylase, can provoke autoimmune responses that contribute to disease progression [95]. CMV infection has additionally been linked to decreased hippocampal volume and abnormalities in the dentate gyrus, structural changes frequently reported in patients with schizophrenia [80,96,97]. These neuroanatomical alterations might be responsible for the cognitive dysfunctions seen in CMV-seropositive individuals, including deficits in memory capacity, processing efficiency, and executive control [181,194,198].

On the other hand, serological and CSF studies provide further support for the implication of CMV in schizophrenia. Elevated CMV IgG and IgM antibodies have been observed in both serum and CSF of schizophrenia individuals, suggesting possible intrathecal antibody production and localised central nervous system infection [194,197,199,200,201,202]. On the other hand, several investigations have shown that CMV seropositivity is linked to more severe neurocognitive impairments and negative clinical features, particularly in female subjects [198,203], although these findings are not universally replicated [132,161,204]. Inconsistency in serological data can reflect methodological differences, such as variations in antibody detection assays, or interfering variables like the influence of antipsychotic treatment on immune markers [133,197]. In particular, CMV infection has been linked to an elevated likelihood of suicide in psychiatric populations, particularly when it co-occurs with other infections such as *Toxoplasma gondii*, implicating a cumulative burden of chronic infections in severe mental illness [205].

Beyond serology, genetic and immunological interactions further support the potential role of CMV in schizophrenia. Polymorphisms in immune-related genes, including TNF-α, interleukin-10 (IL-10), and complement C4, have been correlated with increased susceptibility to CMV and increased probability of schizophrenia, suggesting shared pathways in disease aetiology [16,99]. Genome-wide interaction studies have identified specific loci, such as *CTNNA3 rs7902091*, where prenatal CMV exposure may interfere with the offspring’s genotype to influence susceptibility to schizophrenia [100]. The impact of CMV on immunosenescence, evidenced by telomere shortening in CD8^+^ T cells and elevated inflammatory markers, may also contribute to the accelerated ageing phenotype observed in schizophrenia, characterised by early cognitive decline and increased medical comorbidities [206,207]. Furthermore, childhood adversity, a known risk factor for schizophrenia, appears to exacerbate CMV-associated immune dysregulation, potentially amplifying the neuroinflammatory processes that underlie psychotic symptoms [206].

The therapeutic implications of the CMV–schizophrenia link are an area of growing interest. Antiviral agents such as valacyclovir have shown promise in improving clinical manifestations and cognitive function in individuals with HSV/CMV-seropositive schizophrenia [208,209], although the mechanisms remain unclear. Anti-inflammatory treatments, including cyclooxygenase-2 inhibitors such as celecoxib, have demonstrated adjunctive benefits, possibly by mitigating CMV-driven neuroinflammation [210,211,212]. These findings suggest that targeted antiviral or immunomodulatory strategies may be beneficial in a subgroup of individuals with schizophrenia with evidence of CMV infection or elevated inflammatory markers. However, the absence of consistent CMV detection in postmortem brain tissue [197,213,214,215] and the variability in serological associations underscore the need for more rigorous longitudinal studies to establish causality and identify biomarkers for patient stratification.

In summary, while CMV is unlikely to be the sole causal agent in schizophrenia, accumulating evidence supports its role as a contributor in a subset of subjects, particularly those with early neurodevelopmental insults [82], immune dysregulation [16,94], and specific genetic vulnerabilities [100,216]. The virus may act through direct neurotropic effects [90], immune-mediated neuroinflammation [86], or interactions with additional environmental contributors including early-life adversity [206]. Future research should prioritise large-scale prospective studies integrating serology [197], neuroimaging [80], and genomics [100] to clarify the aetiological significance of CMV and explore personalised treatment approaches for infection-associated schizophrenia subtypes. Until then, the relationship between CMV and schizophrenia remains a compelling but incompletely understood avenue in the search for modifiable risk factors in severe mental illness [89,217,218].

## 6. Human Herpesvirus-6 (HHV-6)

HHV-6, originally isolated from individuals with lymphoproliferative disorders and HIV/AIDS, has a high seroprevalence, with studies suggesting that more than 95% of adults carry antibodies against it. This virus comprises two genetically distinct variants: HHV-6A, known for its greater virulence and cytopathic potential, and HHV-6B, the pathogen responsible for exanthema subitum (roseola infantum) [219].

HHV-6 has been repeatedly detected in the human brain in immunocompromised and immunocompetent populations, demonstrating its ability to induce neurotropism and latency [220]. Therefore, both HHV-6A and HHV-6B can reside in a latent state within the central nervous system and are capable of being reactivated, potentially leading to cognitive and behavioural disturbances [221].

The precise mechanism by which HHV-6 penetrates the central nervous system remains incompletely understood, with a proposed route involving a ‘Trojan horse’ mechanism through which the virus exploits increased endothelial and parenchymal permeability during inflammatory states [220,222]. In particular, the route of entry can influence the pathogenic outcome of infection, including variability in immune function and neurological symptoms. On the contrary, the detection of HHV-6 DNA and antigens in nasal secretions, the olfactory bulb, and associated neural structures suggests that the olfactory pathway may represent a plausible direct route of central nervous system invasion [220].

Interestingly, HHV-6 is unique among herpesviruses due to its ability to integrate into host chromosomal DNA through homologous recombination between telomeric repeats at the ends of its genome and host telomeres, which is a mechanism distinct from episomal latency used by other herpesviruses. This chromosomal integration could disrupt subtelomeric gene regulation or telomere stability, potentially affecting neurodevelopmental pathways implicated in schizophrenia [223]. Latency is further characterized by the expression of latency-associated proteins such as U94, which is implicated in the establishment and maintenance of viral dormancy, although it is not essential for integration. Reactivation of latent HHV-6 may occur, often triggered by simultaneous infection with other herpesviruses, like human herpesvirus-7, or by activated T lymphocytes, particularly in mononuclear cells [224]. Furthermore, co-infection with other microbes may further influence the risk of mood and psychotic disorders by facilitating HHV-6 reactivation in a dose-dependent manner [221].

Moreover, HHV-6 integrated into the genome (inherited chromosomally integrated HHV-6, iciHHV-6), occurring in approximately 1% of individuals, adds complexity to the virus’s neuropathogenic potential, as it can undergo reactivation, particularly under immunosuppressive conditions or pharmacological stimuli, and has been implicated in various inflammatory diseases [101,220].

HHV-6 has been increasingly recognised for its involvement in a spectrum of neurological and psychiatric conditions. Beyond its established association with encephalitis, AIDS encephalopathy, epilepsy, chronic fatigue syndrome, progressive multifocal leukoencephalopathy and febrile seizures, HHV-6 has been implicated in the pathophysiology of Alzheimer’s disease and multiple sclerosis [101,220]. Thus, reactivation of latent HHV-6, particularly under immunosuppression, is known to precipitate encephalitic episodes with long-term sequelae and has been detected in various central nervous system cells [220]. Intriguingly, postmortem analyses have revealed elevated levels of HHV-6A/B protein and DNA within the cerebellum of subjects affected by major depressive disorder and bipolar disorder, suggesting a neuropathological role in these conditions [225].

Furthermore, HHV-6 reactivation has been associated with catatonia, a complex neuropsychiatric syndrome historically linked to schizophrenia during the 20th century but now recognised as a possible manifestation of various psychiatric and somatic illnesses, such as schizoaffective disorder, encephalitis, major depressive disorder, autism spectrum disorder, neurological trauma, bipolar disorder, and autoimmune encephalopathies. In particular, HHV-6 has been shown to influence the morphology of Purkinje cells in the cerebellar cortical region, which are commonly implicated in catatonic presentations. Thus, histopathological and molecular analyses of postmortem cerebellar tissue demonstrate that active HHV-6A infection selectively targets Purkinje cells, leading to significant morphological alterations, including reduced soma size. Fluorescence in situ hybridization confirms viral DNA localization within these neurons, while transmission electron microscopy visualizes intact viral particles, directly linking HHV-6A to Purkinje cell damage. Consequently, recurrent episodes of catatonia may be the result of HHV-6 reactivation, potentially triggered by environmental stressors or psychological burden [225]. Additionally, considering the role of the cerebellum in neuropsychiatric regulation and its disrupted connectivity in schizophrenia, these findings underscore a possible aetiological role for HHV-6 in psychotic pathogenesis [221].

Although certain studies did not identify a statistically strong association between HHV-6 infection and schizophrenia [130,131,134,226], other investigations have documented a significant link between the seropositivity of HHV-6 and the disorder [133,227,228]. Furthermore, some investigations failed to detect HHV-6 genomic sequences in brain samples from autopsies of subjects diagnosed with schizophrenia [137,221,229].

Moreover, a recent case report highlighted a possible link between HHV-6 infection and neuropsychiatric manifestations, particularly schizophrenia-like symptoms. In an immunocompetent middle-aged patient initially diagnosed with schizophrenia, HHV-6 meningoencephalitis was subsequently identified, suggesting a possible infectious trigger for the psychotic episode. These results highlight the significance of considering viral aetiologies, including HHV-6, in atypical presentations of psychosis [230].

## 7. Human Herpesvirus-8 (HHV-8)

HHV-8, a γ-herpesvirus [231], is recognised as the aetiological agent of Kaposi’s sarcoma in individuals with acquired immunodeficiency syndrome. However, it may also be implicated in various other pathological conditions, including multicentric Castleman disease and primary effusion lymphoma, affecting both immunocompromised and, in some cases, immunocompetent individuals [232].

It demonstrates tropism for lymphoid, endothelial, and epithelial cells, establishing a latent state within B lymphocytes. Furthermore, it possesses the capability to infect nerve cells, serving as a harbour for persistent latent infection [232]. Consequently, findings indicating the presence of HHV-8 in the nervous system suggest its potential for neuroinvasion [231]. Recent research has explored the correlation between HHV-8 infection and various neurological diseases, including amyotrophic lateral sclerosis and multiple sclerosis. Thus, the neurotropic nature of this virus has been substantiated by studies detecting HHV-8 DNA in the cerebral tissue of patients with multiple sclerosis [232]. On the other hand, HHV-8 mRNA has been found in the cerebral tissue of individuals with schizophrenia [233], as well as in brain samples from healthy subjects [231]. Moreover, Hannachi et al. reported a significantly higher prevalence of HHV-8 in schizophrenic patients (28.7%) compared to matched controls (14.8%) (*p* = 0.01), with no confounding by sociodemographic or exposure variables [231].

HHV-8 shares numerous biological characteristics with other human herpesviruses, including its phenotypic structure and the presence of genes that encode proteins essential for the two major stages of its life cycle: latent and active replication. However, HHV-8 possesses an exclusive group of genes lacking in other HHVs. Notably, it harbours a significant number of homologs of human host genes, including those encoding chemokine analogues, which are suggested to have originated from the human cellular genome. The genetic diversity of HHV-8 is critically involved in the development of human disorders and malignancies, allowing the virus to invade and modulate its host through multiple mechanisms [234].

Multiple investigations have indicated an increased rate of HHV-8 infection in individuals diagnosed with schizophrenia relative to control cohorts. Furthermore, this infection may be linked to the manifestation of positive symptoms of this disorder [231]. Moreover, HHV-8 has been shown to regulate immune responses, promote cytokine production, and facilitate the selective activation of T-helper type 2 (Th2) lymphocytes [234]. This process can result in the dysregulation of Th1/Th2 cytokine balance, marked by a transition to the Th2 system, which has been proposed as a potential determinant in the development of schizophrenia [235,236].

**Figure 3:**
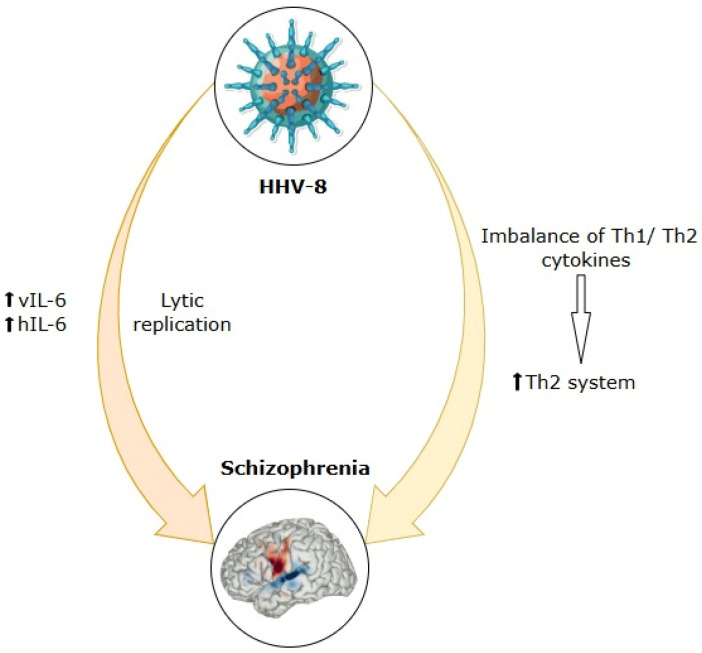
Mechanistic insights into HHV-8 involvement in the aetiopathogenesis of schizophrenia. HHV-8: human herpesvirus-8; vIL-6: viral interleukin-6; hIL-6: human interleukin-6; Th1: T-helper type 1 lymphocyte; Th2: T-helper type 2 lymphocyte; (↑): stimulation; (↓): induction.

Additionally, further evidence supporting the link between HHV-8 and schizophrenia is the fact that the HHV-8 genome expresses a viral variant of interleukin-6 (vIL-6), which exhibits morphological and operational similarities to human interleukin-6 (hIL-6), a cytokine acknowledged to be implicated in the development of schizophrenia [231]. Therefore, it has been shown that vIL-6 exhibits nearly 25% structural similarity to its human equivalent, hIL-6 [237]. Furthermore, HHV-8 lytic replication stimulates the production of both vIL-6 and hIL-6 [238,239] (see Figure 3). As a result, the replication of HHV-8 during initial infection, as well as during reactivation, can produce an impact similar to or even greater than that of hIL-6 in the development of mental disorders [240]. Regarding hIL-6, it is identified as a state-dependent biomarker of schizophrenia, with its levels rising during acute disease exacerbations and being associated with variations in overall psychopathological scores [169].

In conclusion, HHV-8 infection can contribute to the aetiology of schizophrenia through cytokine alterations. Therefore, immune-mediated and pro-inflammatory responses to HHV-8 infection could predispose individuals to the onset of this disorder. Furthermore, these inflammatory processes may re-emerge during HHV-8 reactivation, thereby intensifying disease severity and facilitating the development of positive symptoms [231].

On the other hand, negative and positive symptoms appear to be associated with distinct pathophysiological mechanisms, although these processes may be interrelated. Similarly, the negative symptomatology observed in schizophrenia has been reported to stem from various forms of neural injury that can disrupt other underlying pathophysiological processes [241]. This is why the association of HHV-8 with positive symptoms highlights that the involvement of this virus in schizophrenia is more closely linked to immune mechanisms than to neuronal damage [231].

## 8. Influenza Virus

The hypothesis that influenza virus infection, particularly during gestation, may contribute to the pathogenesis of schizophrenia has garnered substantial scientific interest. This theory stems from early epidemiological observations, reinforced by mechanistic evidence from animal models and molecular studies, and remains a subject of both validation and controversy within psychiatric research. Historical interest in the infectious origins of psychosis dates back to von Economo’s post-World War I descriptions of lethargic encephalitis, which followed influenza pandemics and presented with behavioural and cognitive symptoms reminiscent of schizophrenia. These clinical observations laid the foundation for the idea that neurotropic or neuroinflammatory consequences of influenza infection might contribute to severe psychiatric outcomes [102,242,243,244].

A central line of epidemiological evidence supporting this hypothesis emerged from birth cohort studies following major influenza pandemics. Seminal work by Mednick et al. [245] demonstrated that individuals whose mid-pregnancy period overlapped with the 1957 influenza A2 outbreak that occurred in Helsinki had a significantly increased risk of developing schizophrenia. Comparable associations were later documented by Limosin et al. [246] and O’Callaghan et al. [242], who identified an elevated incidence of schizophrenia among offspring exposed to influenza during mid-gestation. These findings are biologically plausible, since the second trimester encompasses critical neurodevelopmental processes, including neuronal migration and cortical organisation [242,246].

However, seasonal trends in schizophrenia births, with increased frequency of births during winter and early spring, have been interpreted as indirect evidence of prenatal viral exposure during peak influenza activity [102,247,248]. Serological studies further support a temporal vulnerability, revealing a marginally significant correlation between influenza infection in the mother during the first half of gestation and an elevated likelihood of schizophrenia [249].

Animal models have provided robust support for these associations. Maternal infection with influenza or administration of viral mimics like polyriboinosinic-polyribocytidylic acid during gestation reproduces a range of schizophrenia-relevant phenotypes in offspring. These include behavioural abnormalities like impaired prepulse inhibition and social impairments, structural changes such as reduced hippocampus volume and disrupted cortical lamination, and molecular alterations that affect genes implicated in psychiatric disease [102,250].

Mechanically, maternal immune activation instead of direct foetal infection appears to mediate these outcomes. In the absence of consistent transplacental viral transmission, placental pathology, marked by thrombosis, apoptosis, and immune cell infiltration, has been observed, indicating that maternal inflammation disrupts the development of the foetal brain [102,251]. Among pro-inflammatory mediators, IL-6 plays a pivotal role. Elevated maternal IL-6 levels can effectively initiate behavioural deficits in the offspring, which are reversible with anti-IL-6 antibodies. Moreover, elevated maternal IL-6 levels have been shown to cross the placenta and activate foetal glycoprotein 130 (gp130)-dependent signalling, leading to altered Janus kinase/signal transducer and activator of transcription 3 (JAK/STAT3) pathway activity. This cascade disrupts key neurodevelopmental processes, such as neurogenesis, gliogenesis, and synapse formation, particularly in brain regions involved in cognitive and behavioural regulation. These alterations further contribute to long-term abnormalities in synaptic architecture, neurotransmitter function, and neural circuit organisation [102,251,252]. In contrast, the anti-inflammatory cytokine IL-10 can exert protective outcomes, underscoring the importance of cytokine balance in foetal neurodevelopment [252].

Gene expression studies in the foetal and postnatal brains of exposed animals have revealed persistent dysregulation of pathways associated with inflammation, hypoxia, apoptosis, and synaptic plasticity. These include key schizophrenia-associated genes such as FYN proto-oncogene (Fyn) and glutamate ionotropic receptor AMPA type subunit 1 (Gria1) [102]. Additional molecular evidence links prenatal influenza exposure to transcriptional dysregulation of regulator of G-protein signalling 4 (*RGS4*) and *Mx2* [253,254], and to nuclear factor kappa-light-chain-enhancer of activated B cells (NF-κB)-mediated apoptosis in dopaminergic neurones, reflecting dual dopaminergic abnormalities, striatal hyperdopaminergia and prefrontal hypodopaminergia, proposed in schizophrenia pathophysiology [255].

Proteomic and immunogenetic findings have further implicated molecular mimicry and the disruption of proteostasis in the risk associated with influenza. Viral proteins such as hemagglutinin and matrix protein 1 exhibit peptide sequence homology with schizophrenia-related host proteins, potentially triggering cross-reactive immune responses [256,257]. In particular, influenza-homologous peptide sequences are significantly over-represented at the loci of schizophrenia genome-wide association studies, with odds ratios up to 36. Influenza infection also induces aggregation of the Disrupted-in-Schizophrenia 1 (DISC1) protein in animal and cellular models, highlighting the role of impaired protein clearance and misfolding in disease development [258].

Beyond prenatal effects, postnatal exposure to influenza can also confer risk by activating the kynurenine pathway, which produces kynurenic acid, an internal NMDA receptor antagonist involved in glutamatergic dysfunction and dopaminergic imbalance of schizophrenia [259,260,261].

Despite these converging lines of evidence, the influenza–schizophrenia hypothesis is not without challenges. Several large epidemiological studies, including analyses of the 1957 pandemic, have failed to consistently replicate the heightened likelihood of schizophrenia following maternal influenza infection during pregnancy [243,262]. A study paradoxically documented a diminished probability associated with exposure in early pregnancy [263]. A comprehensive systematic review attributed these inconsistencies to methodological variability, including differences in exposure classification, infection timing, and diagnostic criteria [264].

Biological specificity is also debated. Although highly pathogenic influenza strains such as hemagglutinin type 5 and neuraminidase type 1 (H5N1) can cross the placenta [265], most seasonal strains do not exhibit transplacental transmission [102]. This supports the view that neurodevelopmental disturbances are likely mediated by systemic maternal inflammation rather than direct foetal infection. This interpretation is complicated by the observation that physical anomalies associated with schizophrenia often originate in the first trimester, while influenza-related risk is typically concentrated in the second [266]. Sex-specific effects, such as a female-predominant shift from affective to schizophrenic disorders following prenatal infection, add further complexity [267].

Alternative explanations for the seasonal birth effect have also been proposed. The maternal–foetal chronobiological hypothesis posits that reduced maternal melatonin during winter alters foetal circadian signalling, leading to altered striatal and hippocampal development independently of viral exposure [247,248]. Furthermore, noninfectious environmental factors such as prenatal vitamin D deficiency [102,268] and maternal hyperthermia [269] can contribute to neurodevelopmental disruption. Other pathogens, including CMV, herpesviruses, rubella, and *Toxoplasma gondii*, may produce similar outcomes, complicating causal attribution to influenza alone [51,102].

Experimental investigations in adult mice have demonstrated that peripheral influenza infection, even with non-neurotropic strains, leads to sustained hippocampal neuroinflammation. This is characterised by microglial activation, elevated inflammatory mediators, including IL-1β, IL-6, TNF-α, and interferon-alpha (IFN-α), and reduced neurotrophins such as BDNF and nerve growth factor (NGF). The resulting impairments in hippocampal structure and function affect spatial learning and memory tasks [270,271]. Disruption of neurone–microglia communication via reduced CD200 and C-X3-C motif chemokine ligand 1 (CX3CL1) signalling amplifies these inflammatory effects, suggesting long-term vulnerability [270].

Prenatal exposure models further demonstrate that influenza infection during early gestation induces widespread transcriptional changes in both the placenta and the brain of the foetus, including genes related to myelination (for example, myelin basic protein), synaptic signalling (for example, glycine receptors), and cellular stress responses (for example, heat shock protein 70), with persistent effects into adulthood [102,272]. These molecular disturbances are consistent with the observed loss of dopaminergic neurones via NF-κB signalling [255] and receptor imbalances that affect the expression of 5-hydroxytryptamine receptor 2A (5-HT2A) receptors and metabotropic glutamate receptor 2 (mGlu2) receptors [273]. Some models even report infection-induced autoantibodies against NMDA receptors, similar to autoimmune encephalitis, reinforcing the hypothesis of infection-triggered autoimmunity in schizophrenia [244].

However, not all exposed offspring develop schizophrenia-like phenotypes, highlighting the importance of genetic susceptibility and resilience factors. In fact, behavioural deficits in murine models often emerge post pubertally, paralleling the developmental timing of the onset of schizophrenia in humans [273].

Preventive strategies informed by these findings are beginning to gain traction. Influenza vaccination during pregnancy appears safe and may reduce risk without increasing psychosis incidence [244]. Dietary supplements including omega-3 fatty acids, anti-inflammatory interventions, and antiviral agents also warrant further study [273]. Notably, research suggests that anti-inflammatory diets such as the Mediterranean and Dietary Approaches to Stop Hypertension (DASH) patterns—rich in fish, fruits, vegetables, and nuts—may benefit individuals with schizophrenia through multiple mechanisms. These diets help reduce inflammatory cytokines (IL-6, TNF-α), which are implicated in neurotransmission dysfunction, while also supporting gut microbiome balance through prebiotics and probiotics. Additionally, they enhance antioxidant defences, counteracting neural oxidative stress. Although these findings are promising, inconsistencies persist across studies due to variations in study design and socioeconomic barriers that hinder dietary adherence in this population [274]. Promising preclinical data suggest that early antipsychotic treatment may prevent post-pubertal emergence of schizophrenia-like behaviours in exposed offspring [273]. Human induced pluripotent stem cell models offer a promising approach for studying neuroimmune interactions and developing individualised risk assessments [244].

In conclusion, prenatal influenza virus infection is a plausible environmental risk factor for schizophrenia, acting primarily through maternal immune activation, cytokine dysregulation (especially IL-6), molecular mimicry, epigenetic modifications, and disruption of placental–foetal signalling. Although preclinical models provide strong mechanistic support, epidemiological findings remain inconsistent, which requires cautious interpretation. Future research should focus on precisely timed serological and genetic studies, the identification of biomarkers of vulnerability, and the development of early intervention strategies. The interplay between infectious, genetic, and chronobiological influences offers a comprehensive framework to understand the complex aetiology of schizophrenia and to design strategies to mitigate risk [102,247,268,273].

## 9. Hepatitis B and C Viruses (HBV and HCV)

Over the past few years, viral hepatitis has garnered growing scientific attention for its possible involvement in the development of neuropsychiatric conditions [125,275,276,277].

A comprehensive epidemiological investigation involving 91,637 individuals with initial hospital admissions for mood disorders revealed that a past record of hospitalisation due to hepatitis was correlated with a 2.8-fold higher likelihood of mood disorder onset, following a dose–response pattern [278]. Seroepidemiological evidence has also indicated a potential association between severe psychiatric conditions and HBV infection [125], while HCV is recognised to induce a spectrum of non-hepatic clinical features like renal pathology, cardiovascular disease, hypolipidemia, mixed cryoglobulinemia, obesity, and diabetes mellitus, as well as neurological and psychiatric complications [275]. Moreover, HCV infection, independent of interferon-based treatment, has been suggested to contribute to the onset of major depressive episodes, with neuropsychiatric symptoms being observed in nearly half of individuals with chronic HCV infection, regardless of liver disease gravity [277].

Additionally, HCV infection has been linked to an elevated probability of Alzheimer’s disease through its role in sustaining chronic inflammation, which precipitates neuropsychological disturbances and cognitive decline. HCV primarily targets monocytes/macrophages that traverse the blood–brain barrier, culminating in heightened cytokine production and subsequent excitotoxic effects in the central nervous system. Furthermore, HCV viremia is correlated with microglial activation and disrupted cerebral metabolic processes. In particular, elderly individuals with HCV exhibit a greater susceptibility to Alzheimer’s disease, whereas those undergoing antiviral therapy seem to exhibit a lower likelihood of developing the disease [279].

The relationship between schizophrenia and HCV infection has been the focus of research, although the findings have been inconsistent [280]. Consequently, several investigations have proposed that HCV infection may influence the likelihood of developing schizophrenia [281]. Furthermore, although two investigations conducted in the USA identified a significant correlation between schizophrenia and hepatitis C in military veteran cohorts [280,282], the research conducted in Taiwan [283] and Belgium [284] did not corroborate this relationship. Individuals diagnosed with schizophrenia appear to exhibit a heightened vulnerability to infectious hepatitis, with several studies reporting an approximately three-fold rise in the likelihood of hepatitis B and C among this population [285]. These outcomes are corroborated by additional research indicating a higher prevalence of hepatitis in individuals with schizophrenia compared to the general population [286]. Additionally, data from the United States revealed a markedly higher rate of HCV infection among subjects with schizophrenia (16.5%) relative to the overall population (1.9%) [280]. Similarly, the findings by Nakamura et al. revealed a prevalence of HCV of 6.2% in people with schizophrenia, in comparison with 1.2% in the control group, with particularly elevated rates observed among patients over 60 years of age [287]. Comparable rates of HCV infection in individuals with schizophrenia have been documented in the United States (7.1% and 8.2%) [282,288], all notably higher than the estimated global prevalence of HCV (2.8%) [289].

On the contrary, other studies have not observed considerable discrepancies in HCV prevalence between individuals with schizophrenia and the general population [290].

Therefore, despite numerous studies indicating an elevated frequency of HCV infection among individuals with schizophrenia, the evidence is not conclusive. This inconsistency may be attributed to confounding variables such as high rates of comorbid use of intravenous substances and advanced age in this group of patients [275]. Additionally, engagement in high-risk sexual behaviours has been proposed as a causal factor, with individuals with schizophrenia demonstrating over twice the risk for early sexual debut, sexual activity within the past year, and cumulative exposure to sexually transmitted infections [291]. Another plausible explanation involves the dysregulation of immune function in individuals with schizophrenia, rendering them more susceptible to viral infections. This hypothesis is supported by findings of significantly diminished natural killer cell activity, an essential component of innate antiviral defence, among individuals with schizophrenia [292].

**Figure 4:**
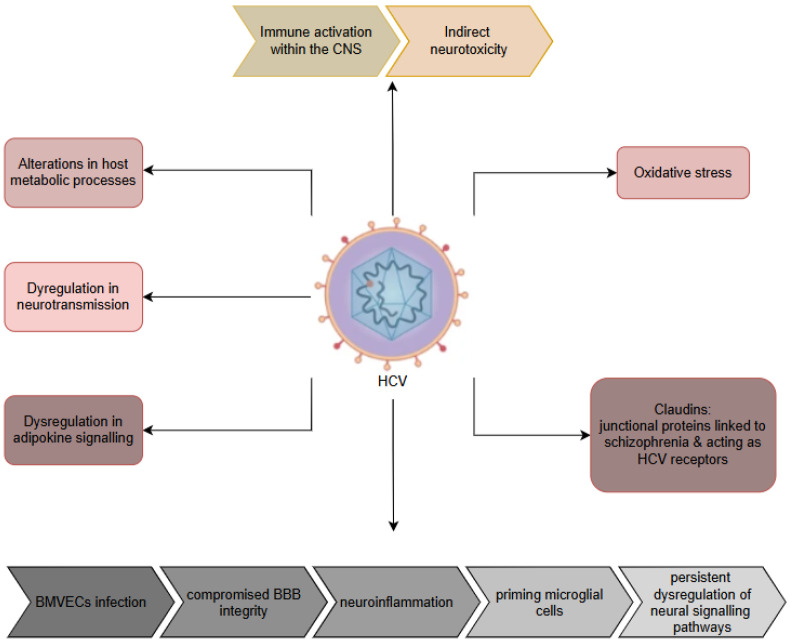
Pathophysiological pathways implicating hepatitis C virus in development of schizophrenia. CNS: central nervous system; HCV: hepatitis C virus; BMVECs: brain microvascular endothelial cells; BBB: blood–brain barrier; QUIN: quinolinic acid.

Regarding the underlying pathways by which HCV infection may lead to the pathogenesis of schizophrenia, it is well established that HCV induces profound alterations in host metabolic processes, neurotransmitter systems, immune function, adipokine regulation, and oxidative stress profiles. Given that inflammation, dysregulation of neurotransmission, metabolic homeostasis, oxidative stress, immune responses, and adipokine signalling are recognised contributors to the onset of schizophrenia, these HCV-induced changes can elucidate the underlying biological connection between the virus and the disorder [275] (see Figure 4).

Additionally, HCV has been shown to elicit immune activation within the central nervous system, possibly resulting in indirect neurotoxicity and nerve tissue injury [278]. Moreover, circulating HCV virions may invade brain microvascular endothelial cells, which are essential constituents of the blood–brain barrier. Viral invasion of these cells often results in compromised stability of the blood–brain barrier, enabling the uncontrolled recruitment of peripheral immune cells into the brain parenchyma, thus promoting neuroinflammation [117].

This inflammatory milieu may, in turn, prime microglial cells, leading to persistent dysregulation of neural signalling mechanisms that are potentially involved in the development of schizophrenia [278].

Although specific adhesion molecules including cell adhesion molecule L1-like (CHL1), neural cell adhesion molecule 1 (NCAM1) and junctional proteins such as claudin 5 (CLDN5) and gap junction protein alpha 8 (GJA8) associated with schizophrenia are not directly involved in the HCV replication cycle, these molecular families are critically involved in microbial recognition and intercellular communication. In particular, claudins serve as entry receptors for HCV, highlighting a potential mechanistic interface between viral infection and neuropsychiatric vulnerability [25].

Interestingly, a community-based longitudinal study conducted between 2003 and 2012 demonstrated that HCV seropositivity was independently linked with an elevated likelihood of developing schizophrenia and that this risk can be mitigated by interferon-based antiviral therapy. Furthermore, among the three cohorts examined, the group of HCV-infected individuals who did not receive treatment exhibited the greatest 9-year cumulative occurrence of schizophrenia, while the cohorts that received antiviral treatment and those not infected with HCV showed comparable rates of incidence of schizophrenia. However, it is still uncertain if the reduction in schizophrenia risk correlated with HCV treatment is a generalisable effect across all antiviral regimens, including direct-acting antivirals, or if it is specific to interferon-based therapies, an issue warranting further investigation [275].

Therefore, in the context of the current direct-acting antivirals era, prioritising the eradication of HCV infection is essential, and antiviral therapy should be universally recommended for individuals with HCV to decrease both liver-related and extrahepatic manifestations, including possible neuropsychiatric conditions such as schizophrenia [275]. To date, no randomized clinical trials have assessed the efficacy of direct-acting antivirals in reducing schizophrenia symptoms. Nonetheless, indirect evidence points to a potential benefit through attenuation of HCV-related neuroinflammation, implicated in cognitive and psychiatric disturbances. For instance, studies reported improvements in memory, attention, and mood in HCV patients’ post direct-acting antivirals therapy, especially among non-cirrhotic individuals. However, the absence of schizophrenia-specific trials limits any firm conclusions. Further studies are needed to determine whether viral clearance via direct-acting antivirals benefits patients with comorbid schizophrenia [275,276].

## 10. Human Immunodeficiency Virus (HIV)

Infection with HIV, another globally widespread pathogen, has been progressively recognised as a driver of chronic psychotic disorders, including schizophrenia, and acute psychotic episodes [21,293,294].

Therefore, the reported incidence rates of the onset of psychosis associated with HIV vary substantially, ranging from 0.23% to 15%, with manifestations typically occurring during advanced stages of HIV infection or during progression to acquired immunodeficiency syndrome (AIDS) [295]. Furthermore, the seroprevalence rates among individuals with schizophrenia have been reported to exceed those of the general population [296]. Although the prevalence of HIV in schizophrenia cohorts in the USA (estimated at 0.6% according to WHO data) is generally greater than that observed in the overall population, the reported rates vary widely between 1.3% and 22.9%, with lower rates observed in regions such as Asia [286].

Further evidence supports the notion of retroviral antigen involvement, specifically HIV-1-related antigens in subgroups of individuals with a spectrum of schizophrenia and bipolar disorders. Hart et al. employed HIV-1 Western blot assays to screen psychiatric patients, revealing that 52% exhibited serum reactivity to at least one HIV-1 antigen (primarily gag proteins p24 and p17). This reactivity, distinct from HIV-1 infection, was interpreted as potential exposure to antigenically related retroviruses, given the assay’s cross-reactivity with conserved retroviral epitopes. Therefore, these findings underscore a possible retroviral link in neuropsychiatric pathophysiology, warranting further investigation into viral mimicry or endogenous retroviral activation [297].

Beyond psychosis, HIV infection has been causally linked to several well-characterised psychiatric syndromes, including major depressive and anxiety disorders, likely attributable to the virus’s capacity to replicate within the central nervous system [278,298]. HIV also affects the peripheral nervous system. Thus, between 30% and 60% of individuals diagnosed with AIDS develop HIV-related sensory neuropathy, typified by the degeneration of both myelinated and unmyelinated nerve fibres [117]. On the other hand, the progression of HIV to AIDS is associated with a probability of nearly 50% neurological impairment, from peripheral neuropathies to frank dementia [82].

In addition, HIV-associated neurocognitive disorders are frequently observed in HIV-positive individuals [298]. Despite advancements in combination antiretroviral therapy, many HIV-positive individuals, especially those with well-controlled viral loads, continue to experience HIV-associated neurocognitive disorders. Neuroimaging studies have shown a decrease in cortical thickness and subcortical volumes in such individuals relative to matched controls, although longitudinal assessments indicate that combination antiretroviral therapy can decelerate the progression of neurodegeneration. Furthermore, studies using Tat-transgenic mouse models implicate the HIV-1 transactivator of the transcription (Tat) protein in morphological brain alterations, encompassing ventricular dilation and cortical thinning, accompanied by reductions in synaptic markers, thus supporting a neurodegenerative role for Tat independent of viral replication [121].

However, HIV-associated dementia, the most severe neurocognitive complication of HIV, affects up to 30% of the infected population and is marked by pronounced neuronal loss, microglial nodules, multinucleated giant cells, and reactive astrocytosis. Commonly affected brain areas, including the hippocampus and substantia nigra pars compacta, are also implicated in Alzheimer’s disease and Parkinson’s disease. Although the precise mechanisms of HIV-associated dementia remain incompletely understood, the proposed contributors include neurotoxicity of viral proteins, cytokine-mediated inflammation, and macrophage-driven HIV replication in the central nervous system. Proteomic analyses of HIV-associated dementia patients have identified 31 significantly dysregulated proteins, implicating mechanisms associated with axonal pathfinding and mitogen-activated protein kinase signalling that regulate neurodegenerative processes in Alzheimer’s disease and Parkinson’s disease [279].

Moreover, investigations have shown that HIV-positive individuals, even in the absence of significant neuropathological alterations, exhibit elevated psychiatric morbidity, particularly cognitive dysfunction and a higher prevalence of psychotic disorders, possibly linked to low-grade central nervous system inflammation [299].

Interestingly, using advanced CSF analytical techniques, approximately 40% of people with treatment-resistant schizophrenia have been observed to meet criteria for possible or probable low-level neuroinflammation or mild encephalitis, substantiated by evidence of activated CSF immune cells despite normative cell counts [34]. Furthermore, early manifestations of HIV encephalopathy often include intrathecal synthesis of HIV-specific antibodies and, in some cases, oligoclonal IgG bands, despite normal CSF cell counts and albumin quotient. This diagnostic ambiguity presents challenges in addressing low-level chronic infections, as highlighted in the hypothesis of mild encephalitis, which also considers viral latency and reactivation as contributing mechanisms [299].

These findings underscore the heterogeneity of schizophrenia, in which low-level neuroinflammation may represent a more prevalent aetiological substrate than previously assumed. A growing body of research implicates various low-virulence pathogens as risk factors for psychotic and autoimmune illnesses. In line with the hypothesis of mild encephalitis, low-level neuroinflammation constitutes a central pathophysiological process in a schizophrenia subset that exhibits syndromic overlap with other psychiatric conditions. Triggers for this inflammatory state can include infections, autoimmunity, environmental toxins, or traumatic injury. The hypothesis further posits that delayed environmental insults (“late hits”) and gene–environment interplay are integral to the development of schizophrenia, consistent with current epidemiological and molecular findings [34].

Clinically, HIV-related psychosis is often characterised by somatic, persecutory, and grandiose delusions, with hallucinations constituting a prominent secondary symptom cluster. The therapeutic impact of highly active antiretroviral therapy on psychotic features remains inconclusive, further confounded by the potential neuropsychiatric side effects of antiretroviral agents, including hallucinatory phenomena. Although antipsychotic medications are generally effective in treating HIV-related psychoses, people living with HIV exhibit an increased susceptibility to extrapyramidal side effects and tardive dyskinesia, particularly when administered first-generation antipsychotics [295].

Meanwhile, vertical HIV transmission remains a significant concern, with transmission rates reaching 25% in untreated mothers. Neonatally infected neonates often exhibit neurological deficits, attributable both to direct viral neuropathology and to secondary opportunistic infections. The neuroinvasive capacity of HIV is complex and is currently understood to involve monocyte-mediated central nervous system infiltration [82].

**Figure 5:**
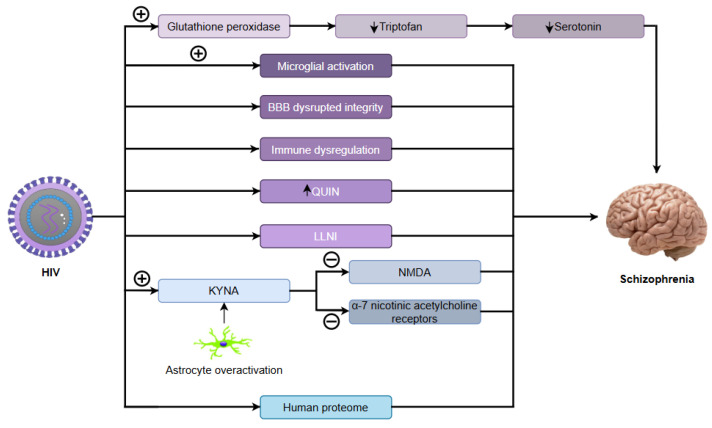
Immunopathological and neurobiological mechanisms connecting HIV and schizophrenia. HIV: human immunodeficiency virus; BBB: blood–brain barrier; LLNI: low-level neuroinflammation; KYNA: kynurenic acid; NMDA: N-methyl-D-aspartate receptor; (+): stimulation; (−): inhibition; (↓): reduction; (↑): increase.

Therefore, lentiviruses such as HIV and simian immunodeficiency virus (SIV), as well as human T cell leukaemia virus (HTLV), can penetrate the central nervous system through a “Trojan horse” process, in which infected monocytes or macrophages traverse the blood–brain barrier. Human immunodeficiency virus predominantly infects CD4^+^ T lymphocytes, but also targets macrophages or microglia, as well as astrocytes, which express CD4 and the requisite chemokine-associated co-receptors. The viral glycoproteins gp120 and gp41 mediate viral entry. The host immune response significantly modulates viral replication; cytokine signalling can enhance or inhibit replication, depending on the cellular context and cytokine profile. Within the central nervous system, virus-carrying memory T lymphocytes and monocyte progenitors cross the blood–brain barrier and transform into perivascular microglial cells, initiating viral replication and cytokine-mediated inflammation that contribute to neurodegenerative processes, including cognitive impairment and mood disorders. Human immunodeficiency virus, human T cell leukaemia virus, and mouse adenovirus 1 (MAV-1) infections may further damage the blood–brain barrier by degrading intercellular junctional proteins. Moreover, immune dysregulation triggered by HIV may enhance the neuropathogenic potential of concurrent viral infections, as observed in co-infections involving HIV and other neurotropic viruses, such as HCV, John Cunningham virus (JC) virus, or HCMV [117] (see Figure 5).

In contrast, microglial reactivity is an essential component in the neuropathology of neurodegenerative disorders, serving as a hallmark of neuroinflammation. As a neurotropic virus, HIV is involved in the maintenance of chronic neuroimmune activation, leading to enduring neuroinflammatory states. The persistent expression of viral proteins within neural tissues is thought to underlie such inflammatory cascades [279]. In neonatal HIV-1 infection, widespread microglial activation and astrogliosis are commonly observed, along with impaired cerebral development. In particular, unlike neonatal bipolar disorder, HIV-infected microglia actively internalise the virus and may support replication in the presence of pro-inflammatory cytokines [300]. Meanwhile, studies conducted in animal models have consistently shown that microglial reactivity is correlated with schizophrenia-relevant endophenotypes, including impaired prepulse inhibition and deficits in working memory. These studies use prenatal and postnatal exposure paradigms that involve pro-inflammatory agents including HIV, polyriboinosinic-polyribocytidylic acid, and GM-CSF. Affected rodents frequently exhibit behavioural phenotypes consistent with schizophrenia together with microglial expansion and activation. Interestingly, these neurobehavioral deficits, along with neuroinflammatory markers, are often reversed through treatment with atypical antipsychotics or microglia-targeting agents such as minocycline [301].

In particular, a substantial proportion of genes implicated in schizophrenia are involved in glutamatergic signalling and postsynaptic density. While only a few of these genes directly interact with viral life cycles, certain proteins, such as FYN and citron kinase, interact with viral components (e.g., herpes simplex and rubella), and others such as Protein interacting with C kinase 1 (PICK1) and nectin receptors modulate glutamate receptor function and serve as viral entry portals. HIV has also been shown to bind to NMDA receptors, suggesting a plausible mechanistic overlap in pathogen–host interactions within the central nervous system [25].

In addition, interactions between HIV-1 and human genes or proteins have been documented, including protein–protein binding, modulation of gene transcription, phosphorylation, and gene expression. To date, 1510 such viral–host interactions have been identified among approximately 29,277 human proteins, indicating that HIV-1 may influence the function of around 5.2% of the known human proteome. In particular, among the 245 genes associated with susceptibility to schizophrenia, a disproportionately high percentage is affected by other pathogens: influenza virus (21%), HSV-1 (22%), CMV (18%), rubella (12.6%), and *Toxoplasma gondii* (16%). These findings point to a possible over-representation of pathogen-responsive genes within schizophrenia-related genetic profiles. Moreover, preliminary analyses reveal that 114 of the 245 candidate genes (46%) are involved in the replication cycles of multiple infectious agents, a proportion likely to increase with further consideration of additional organisms such as *Chlamydia* spp. and BoDV [25].

Emerging evidence implicates quinolinic acid, a harmful neuroactive byproduct in the kynurenine pathway, in neuronal degeneration during central nervous system infections. Postmortem examinations of brain tissue in subjects with schizophrenia have demonstrated elevated microglial concentration and increased quinolinic acid levels. These findings are accompanied by reduced tryptophan levels and upregulated indoleamine 2,3-dioxygenase function in the frontal brain region, suggesting perturbations in the kynurenine pathway. Enhanced concentrations of 3-hydroxykynurenine have been observed in HIV patients, particularly those with dementia or liver encephalopathy, and are associated with biomarkers of immunological stimulation including β2-microglobulin and neopterin [302].

In particular, HIV-positive individuals with psychotic symptoms exhibit significantly higher levels of CSF kynurenic acid than their counterparts without psychiatric manifestations [303]. Moreover, increased concentrations of kynurenic acid in cerebrospinal fluid have also been documented in schizophrenia, with the antagonistic action of kynurenic acid on NMDA receptors and α7-nicotinic acetylcholine receptors implicated in both psychotic and cognitive symptoms [302,303]. Additionally, other studies also indicate elevated kynurenic acid production in cultured fibroblasts of patients with bipolar disorder and schizophrenia, suggesting systemic dysregulation of kynurenine pathway [304]. Astrocytosis overactivation in schizophrenia likely contributes to this accumulation of kynurenic acid, potentially exacerbating the hypofunction of the NMDA receptor, a hallmark of the disorder. In addition, studies have hypothesised that early postnatal elevations in quinolinic acid due to viral infections can precipitate the development of schizophrenia in the adolescent or young adult period. In HIV-infected individuals, concentrations of quinolinic acid in the CSF can be elevated up to 300 times, although zidovudine treatment significantly reduces these levels and correlates with neurological improvement. Similarly, HIV-positive children demonstrate a four-fold increase in quinolinic acid levels, which normalises with zidovudine therapy. These elevations are believed to originate from immune-activated cells responding to systemic viral invasion [302].

Finally, serotonin depletion in AIDS patients, linked to HIV-induced depletion of tryptophan through upregulation of selenoenzyme glutathione peroxidase, can contribute to neuropsychiatric manifestations, including psychosis. This serotonergic deficiency provides another plausible biochemical pathway through which HIV infection can exacerbate or precipitate schizophrenia-spectrum conditions [305]. It is known that HIV-induced serotonin depletion and oxidative stress (via indoleamine 2,3-dioxygenase activation and glutathione peroxidase dysfunction) contribute to psychosis by disrupting glutamatergic and dopaminergic signalling. Therefore, targeting the kynurenine pathway, antioxidant defences, and NMDA receptors may mitigate these effects [294].

However, studies using polymerase chain reaction methods to detect HIV nucleic acids in patients with schizophrenia have produced inconclusive associations between HIV infection and schizophrenia [306].

Meanwhile, recent investigations employing Virobiome-Seq, a new viral metagenomic sequencing approach, have identified HIV-1 genetic material in peripheral blood mononuclear cells, plasma, and faecal specimens from subjects with mental disorders. This technique also discovered numerous uncharacterized circular RNA viruses, though their origins remain ambiguous. These findings underscore the incomplete nature of current virome cataloguing and highlight the potential of Virobiome-Seq to elucidate complex interactions between viruses and psychiatric disorder [125].

## 11. Human Endogenous Retroviruses (HERVs)

HERVs are genomic traces of ancestral retroviral infections which represent approximately 8% of human genetic material. Although normally silenced by epigenetic mechanisms, several HERV families, most notably HERV-W and HERV-K, have been shown to exhibit transcriptional activity in schizophrenia. Numerous investigations have demonstrated elevated concentrations of HERV-W RNA and proteins including envelope protein (Env), group-specific antigen (Gag), and polymerase (Pol) in peripheral blood, CSF, and cortical brain tissues of schizophrenia individuals, while being absent or negligible in healthy controls [307,308,309,310,311,312,313,314]. Antibodies directed against the HERV-W and HERV-K proteins have also been detected at higher levels in patients with recent-onset psychosis, indicating active retroviral expression during early stages of the disease [315,316].

Aberrant HERV expression in schizophrenia appears to be the result of both genetic predisposition and epigenetic dysregulation. Hypomethylation of the HERV-W and HERV-K loci has been identified in individuals with first-episode psychosis, suggesting an early disruption of epigenetic silencing mechanisms that can contribute to disease onset [317,318,319]. These epigenetic changes include DNA hypomethylation and chromatin remodelling at long terminal repeats (LTRs), which facilitate transcriptional activation in neural and immune cells. Genetic evidence further supports this mechanism: for instance, HERV-K elements have been shown to act as enhancers of proline dehydrogenase (*PRODH*), a schizophrenia susceptibility gene involved in glutamatergic transmission [318,320]. Additionally, chromosomal linkage studies have implicated the 22q13 region—home to APOBEC3G, an inhibitor of retroviral replication—as a potential locus of genetic vulnerability to HERV activation [321].

Moreover, HERV-W activation contributes to schizophrenia through robust neuroimmune mechanisms. The env protein of HERV-W acts as a strong inducer of TLR4, activating the NF-κB and JAK-STAT1 signal transduction cascades and leading to the upregulation of pro-inflammatory cytokines including TNF-α, IL-6, and IL-1β [9,307,322,323,324]. HERV-W Env also promotes inducible expression of nitric oxide synthase (iNOS), leading to oxidative stress and nitric oxide-mediated neurotoxicity [9,307,325]. Elevated CRP concentrations and systemic inflammation observed in patients with schizophrenia support a model in which HERV-W expression bridges immune activation with neuronal dysfunction, even in the absence of active infection [307,325]. These processes contribute to the disruption of the blood–brain barrier, microglial activation, and chronic neuroinflammation—hallmarks of the pathophysiology of schizophrenia [9,307,322,323,324,325].

Beyond inflammation, HERV-W and HERV-K directly influence neurodevelopmental and neurotransmission pathways. Therefore, HERV-W env has been proven to modulate the expression of critical neuroplasticity genes, such as BDNF, neurotrophic receptor tyrosine kinase 2 (NTRK2/TrkB), and dopamine receptor D3 (DRD3), all of which are associated with synaptic development, neuronal differentiation, and dopaminergic signalling [308,326]. The Env protein also interacts with the sodium-dependent amino acid transporters human alanine/serine/cysteine/threonine transporter 1(hASCT1) and human alanine/serine/cysteine/threonine transporter 2 (hASCT2), disrupting glutamate uptake and contributing to excitatory/inhibitory imbalance, a central feature of schizophrenia [310,326]. Furthermore, HERV-W ENV downregulates 5-HT4 receptors and activates small-conductance calcium-activated potassium channels 2 and 3 (SK2/SK3), impairing hippocampal long-term potentiation and reducing neuronal excitability—mechanisms directly linked to cognitive deficits in schizophrenia [327,328,329,330].

Interestingly, recent findings have revealed a novel mechanism involving ferroptosis, which shows that HERV-W Env downregulates neuroprotective genes such as glutathione peroxidase 4 (*GPX4*) and solute carrier family 3 member 2 (*SLC3A2*), leading to iron accumulation, lipid peroxidation, and mitochondrial dysfunction. This adds a layer of neurotoxicity mediated by HERVs and supports their involvement in progressive neuronal damage observed in schizophrenia [331].

However, environmental factors play a central role in HERV reactivation, supporting a gene–environment interaction framework. Viral infections such as influenza, HSV-1, HIV, EBV, and *Toxoplasma gondii* can transcriptionally activate HERVs, particularly HERV-W, through inflammatory signalling pathways. Therefore, *T. gondii* and viruses such as HIV, EBV, and HSV-1 synergistically activate HERV-W through converging NF-κB pathways. The parasite establishes chronic NF-κB activation, while viral proteins such as HIV Tat, EBV latent membrane protein 1 (LMP-1), and HSV-1 infected cell protein 0 (ICP0) further stimulate this pathway and directly bind to HERV promoters. This convergence elevates pro-inflammatory cytokines (TNF-α, IL-6, IFN-γ) that both enhance HERV-W transcription and disrupt its epigenetic silencing. The shared use of these inflammatory mechanisms explains how multiple pathogens can collectively amplify HERV-W activation [307,310,311,316,319,324,332,333]. These environmental exposures may serve as the first “hit” in the two-hit theory of schizophrenia, where prenatal immune activation primes the brain, and later life stress or infections lead to full disease expression through HERV reactivation [307,310,311]. Therefore, elevated expression of HERV-W has been observed following infections with *T. gondii*, HSV-1, and influenza virus in both immune and neural cells [311]. Perinatal infections can initiate epigenetic de-repression of HERVs, and these elements can remain transcriptionally active into adulthood, promoting persistent neuroinflammation [307,311]. Importantly, HERV-W expression patterns appear to be independent of general immune cell activation, indicating a specific pathogenic role [311,319]. All these mechanisms regarding the association between HERV and schizophrenia can be seen in Table 3.

On the other hand, HERV-W activation has strong clinical relevance. Detection of elevated HERV-W Env, Gag, and pol transcripts and proteins in plasma, CSF, and postmortem brain tissue from patients with schizophrenia provides potential biomarkers for diagnosis or disease monitoring [308,309,311,313]. Interestingly, in recent-onset schizophrenia, retroviral pol transcripts homologous to ERV9 were identified in approximately 34.5% of patients but were absent in healthy controls, highlighting a specific association with early psychosis [315].

Epigenetic findings also offer therapeutic insight. Therefore, hypomethylation of the HERV loci in patients in the early stages has been shown to normalise after antipsychotic treatment, suggesting that pharmacological modulation of epigenetic status may regulate HERV expression [311,317]. Furthermore, HERV-W ENV-activated inflammatory pathways, such as Toll-like receptor 4 (TLR4), JAK-STAT1, or NF-κB, represent additional drug targets for immunomodulatory or antiviral therapies [307,316,323,324]. On the other hand, shared HERV activation profiles between schizophrenia and bipolar disorder underscore a transdiagnostic mechanism that may explain overlapping clinical features and genetic architecture [312,334,335].

## 12. Zika Virus (ZIKV)

ZIKV constitutes a recently recognized flavivirus and is one of the most extensively investigated pathogens known to disrupt neurodevelopment. While it is transmitted primarily through *Aedes* mosquitoes, alternative modes of transmission, including vertical, sexual, and blood transfusion routes, have likewise been documented. It belongs to the group of single-stranded RNA viruses classified within the Flaviviridae family and the *Flavivirus* genus [336,337].

Research has indicated that neurotropic flaviviruses can exert complex effects on a wide range of neurological processes, implying that these infections may lead to the development or aggravation of neuropsychiatric diseases, such as mood and psychotic illnesses [338]. Therefore, ZIKV exhibits pronounced neurotropism, especially during foetal development [338,339]. Given the neuropathological parallels between congenital Zika syndrome and TORCH-related congenital infections [340], it has been hypothesised that neurodevelopmental disruptions induced by utero ZIKV infection could contribute to the development of adult-onset neurological conditions such as schizophrenia [339].

Although ZIKV is known to cause serious birth defects, including microcephaly when transmitted early in pregnancy, its effects on developmentally normal foetuses exposed later in gestation are unclear [336,338,339]. More studies are needed to explore whether such late exposure could lead to long-term neurodevelopmental and behavioural consequences [338]. Thus, it is proposed that, similar to maternal infections with CMV and influenza A, ZIKV during pregnancy may impair foetal brain development by inducing the discharge of maternal pro-inflammatory mediators, initiating neuroinflammatory responses in the foetal brain, and/or directly targeting foetal neural cells. This viral infection has been observed to mirror the pathogenesis of various neurodevelopmental conditions, being associated with reduced brain volume, disorganisation of the cortical layer, and increased apoptosis within the hippocampus and cortex, along with the loss of neural progenitor cells, which is induced through multiple programmed cell death processes, including apoptosis, pyroptosis, and autophagy [337].

Therefore, preclinical investigations have indicated that neonatal contact with ZIKV may lead to immediate and long-term behavioural impairments in murine models, with viral ribonucleic acid remaining within the brain for a minimum of 100 days [341]. Furthermore, evidence suggests that ZIKV can trigger renewed phases of viral multiplication far beyond the initial acute infection, being identified in placental tissue up to 200 days post infection in pregnant women, and generally about 160 days in the cerebral tissue of infants diagnosed with congenital Zika syndrome. Additionally, ZIKV has demonstrated the capacity to multiply and remain within multiple types of tissue, including cerebral tissue, lymphatic tissue, and testes, indicating its potential to establish latent infections [336]. On the contrary, microglial cells allow for viral replication without undergoing cell death, suggesting a potential role as persistent reservoirs within the foetal brain. Given their origin in the yolk sac, microglia likely facilitate viral entry through the maternal circulation and subsequently disseminate ZIKV throughout the central nervous system. Additionally, they initiate a sustained neuroinflammatory response through the discharge of pro-inflammatory mediators such as IL-6, IL-1β, TNF-α, and Monocyte Chemoattractant Protein-1 (MCP-1) [337].

Recovery from neuroinvasive flavivirus infections, including ZIKV, is often linked to cognitive impairment due to immune-mediated synaptic dysfunction and disrupted neurogenesis. These observations have been noted in both preclinical models and human patients, where persistent memory loss and cognitive decline are commonly reported [338]. Moreover, studies have shown that a significant percentage of neonates with ZIKV infection, who present structural cerebral anomalies at birth, are likely to experience behavioural, cognitive, and psychiatric challenges in adulthood [342]. Additionally, the most characteristic behavioural consequences of neuroinvasive flavivirus infection in animal models involve alterations in cognitive functions, particularly learning and memory [338].

However, asymptomatic prenatal infections with ZIKV could exert persistent influences on neurodevelopment. Therefore, studies using neural organoids originating from embryonic stem cells have shown that ZIKV infection during foetal development may contribute to delayed-onset neuropsychiatric conditions by modifying the DNA methylation patterns of differentiated neurones, astrocytes, and neural progenitors, which could ultimately influence the regulation of essential genes involved in various neuropsychiatric diseases, such as schizophrenia and intellectual impairment [343]. Therefore, ZIKV infection modulates the expression of more than 40 genes implicated in neuronal differentiation, apoptosis, synaptic transmission, and neurogenesis [338,339].

Modifications in neurological gene expression were also identified within the central nervous system in vivo by applying multiple immunocompetent murine models of ZIKV infection. Among the regulatory pathways implicated, TNF-α signalling through tumour necrosis factor receptor 1 (TNFR1) has been identified as a key mechanism that mediates ZIKV-induced modifications in neural gene expression. The evidence linking ZIKV to schizophrenia is supported by transcriptomic data showing dysregulation of psychiatric disorder-associated genes, including those implicated in schizophrenia, such as nuclear receptor subfamily 4 group A member 2 (*NR4A2*) and neuroligin 1 (*NLGN1*), following ZIKV infection. The role of TNF-α signalling is robustly demonstrated in vitro and in vivo. Specifically, it was shown that ZIKV infection in neurons upregulates TNF-α, which in turn alters the expression of psychiatric disorder-related genes, while TNFR1 blockade rescues ZIKV-induced gene expression changes. However, while these findings highlight a plausible mechanistic pathway, their direct relevance to human schizophrenia remains speculative, as clinical studies correlating ZIKV infection with psychiatric outcomes are limited. Further human epidemiological and molecular studies are needed to validate these associations [338].

On the other hand, ZIKV appears to preferentially target neural progenitor cells via Toll-like receptor 3 (TLR3) signalling, leading to downregulation of specific genes [337]. The finding that methylation alterations are notably pronounced in a heterogeneous organoid-based environment compared to isolated neuronal cells indicates that both the virus and the broader cellular and immunoreactive context influence DNA methylation. However, it remains unclear whether these methylation modifications precede viral infection or whether the infection disrupts epigenetic regulatory genes before any epigenetic changes occur [343].

Meanwhile, additional research has indicated that ZIKV infections in adolescents can be related to the onset of psychotic disorders [344] and severe depressive episodes [345]. On the contrary, several case reports have revealed the emergence of psychotic manifestations, such as hallucinations, in individuals infected with ZKV [344,346].

The main argument supporting a potential link between ZIKV infection and certain psychiatric diseases, including major depressive disorder and schizophrenia, is related to neuroinflammatory processes and the impact of the pronounced response of innate cytokines to ZIKV infection on behaviour [338]. On the other hand, several studies [347,348], though not all [349], have identified developmental delays or impairments that manifest before the age of three. These observations on early childhood neurodevelopmental deficits linked to ZIKV exposure are noteworthy, as schizophrenia is similarly characterised by premorbid general cognitive impairments [350].

In conclusion, although current data are insufficient to establish a direct connection to schizophrenia, long-term epidemiological research is crucial to determine the hypothesized contribution of ZIKV in neuropsychiatric outcomes such as schizophrenia [351].

## 13. Borna Disease Virus (BoDV)

Borna disease virus 1 (BoDV-1), a negative-sense, single-stranded RNA virus with an enveloped structure, belongs to the Bornaviridae family and is the pathogen responsible for Borna disease, a fatal neurological condition affecting both domestic animals and humans. This pathology is attributed to zoonotic transmission originating from its natural host, *Crocidura leucodon* [352,353]. Therefore, as of mid-2024, approximately 50 molecularly confirmed human BoDV-1 cases have been documented, predominantly presenting as fulminant encephalitis [354].

BoDV-1 is characterised by its unique replication within the host cell nucleus, distinguishing it from other members of the *Mononegavirales* order, which typically replicate in the cytoplasm. The virus exhibits neurotropism, particularly in the limbic system, and can persist in neuronal tissue without inducing cytolytic effects [353]. Moreover, BoDV-1 appears to act as an endozoonotic agent in numerous vertebrate hosts, leading to chronic and often latent central nervous system infections, particularly targeting the hippocampus [101].

The viral tropism for the limbic–hypothalamic axis and the observation of affective disorder-like syndromes in infected animal models support its candidacy as a neurotropic contributor to human psychiatric disorders, with prenatal BoDV infection being considered a potential contributor to the risk of schizophrenia [18,355].

Thus, neonatal exposure to BoDV in rodent models has been demonstrated to induce impaired prepulse inhibition, impaired startle response habituation and heightened sensitivity to stimulants, mimicking core features of schizophrenia and responding to antipsychotic therapy [355,356]. Such infections also induce gliosis, indicating neurodegenerative processes [355]. Moreover, experimental infection in animal models has induced neurobehavioral changes such as anxiety and aggression, with histopathological findings of hippocampal dentate gyrus degeneration in advanced stages. These results should be carefully contextualized when considering implications for schizophrenia, as important limitations exist. Notably, rodent models fail to capture both the disorder’s intricate gene–environment interplay and cross-species variations in neuroinflammatory pathways that could modify pathological outcomes. Although informative for understanding basic mechanisms, human studies remain essential to verify these findings’ applicability to schizophrenia [101].

Furthermore, BoDV-1 infection in the central nervous system of animals has been associated with sporadic brain-related presentations, comprising meningitis, encephalitis, motor abnormalities, and, in certain cases, psychiatric symptoms analogous to schizophrenia [357]. In addition, BoDV-1 infection during neurodevelopmental stages, particularly in cerebellum Purkinje cells, parallels neuropathological findings in both schizophrenia and autism models [300,358,359].

On the contrary, neonatal infection is characterised by a persistent viral presence with minimal inflammation, hippocampal neurodegeneration, particularly dentate granule cells, and deficits in spatial learning [7]. These animals also show growth retardation, disruptions in sleep–wake cycles, reduced play behaviour, impaired development motor coordination, and altered neurodevelopmental trajectories [300].

In addition, intracerebral BoDV injection in animal models results in neuronal loss within the hippocampus, cerebellum, and neocortex, along with behavioural disturbances such as hyperactivity and social withdrawal. These effects correspond to dynamic changes in brain cytokine profiles [360]. Moreover, persistent infection with BoDV, viral reactivation, recurrence of symptoms, and chronic pathological outcomes have been documented in animal models [34].

The heterogeneous progression of schizophrenia has been seen as comparable to that of autoimmune conditions, implicating a triad of genetic, environmental, and immunological factors. Within this framework, BoDV-1 has been proposed as a potential aetiological agent in a rare subtype of schizophrenia characterised by mild encephalitis features [34]. Interestingly, patients diagnosed with mood and psychotic disorders demonstrated a significantly higher rate of identification of BoDV in peripheral blood mononuclear cells (33.3%) compared to healthy controls (13.3%) [361].

Therefore, the elevated levels of seroprevalence in patients with mental disorders such as schizophrenia, depression, and autism have led some authors to propose a potential connection between BoDV-1 and chronic psychiatric illnesses, with evidence of neurotransmitter disruptions that could underlie psychiatric symptomatology [362,363,364,365]. In particular, chronic noncytolytic BoDV infection has been associated with neurotransmitter imbalances, particularly with dopamine and serotonin, and broader cellular dysfunctions [34]. Therefore, initial serological investigations in the 1980s identified anti-BoDV antibodies in patients with mood disorders, prompting hypotheses on its potential aetiological role in psychiatric disease [366].

Meanwhile, various serological studies have reported increased seropositivity to BoDV-1 and persistent BoDV-1 markers among individuals with psychiatric disorders, particularly those diagnosed with schizophrenia, bipolar disorder, and severe depressive disorder, raising the hypothesis of chronic subclinical infection contributing to the pathophysiology of the disease [130,353,367,368,369]. Multiple investigations have reported statistically relevant correlations between BoDV infection and schizophrenia, with higher BoDV seropositivity observed in individuals with schizophrenia relative to the general population, supporting the hypothesis that the affinity of the virus for limbic structures may be the basis for its proposed role in disease aetiology [130,368,369,370,371,372].

On the other hand, serological evidence from certain studies has demonstrated elevated levels of BoDV-specific antibodies in individuals with schizophrenia, particularly those with negative symptomatology [373,374]. Moreover, when comparing psychiatric patients based on the presence or absence of antibodies, those who were positive exhibited a higher prevalence of first-episode schizophrenia without full remission, along with a greater incidence of suicide attempts [375]. In addition, a case of first-episode schizophrenia showed elevated BoDV-specific serum antibodies, detected via an indirect immunofluorescence assay, over the initial five weeks of hospitalisation [376].

On the contrary, another study analysed anti-BoDV-1 antibody titres using an indirect immunofluorescence assay in psychiatric patients who underwent repeated testing and observed that individuals with schizophrenia in later stages exhibited higher antibody levels compared to those in earlier stages. The authors proposed that BoDV-1 infection may persist in individuals with schizophrenia who consistently demonstrate specific seropositivity [364].

Detection of BoDV-1 RNA in individuals with schizophrenia and schizoaffective disorder, as well as in their relatives, has also shown higher positivity rates compared to controls [18]. The reported seroprevalence among patients with schizophrenia ranged between 3% and 45%, in contrast to 0–5% in non-psychiatric populations [377]. Furthermore, an electroluminescence-based assay that detects BoDV-1 proteins p40 and p24 in human serum revealed a significantly higher prevalence of infection among schizophrenic patients [367]. Conversely, some investigations using Western blotting or RT-PCR yielded inconsistent results, suggesting methodological variability [378,379,380,381]. Consequently, among studies examining BoDV p24 antibodies and RNA in patients with schizophrenia, some reported elevated levels compared to healthy controls, while others did not confirm such associations [382,383,384].

However, although the identification of BoDV RNA or proteins in human specimens has produced inconsistent results, some investigations have revealed the detection of BoDV sequences in the brains of patients with affective and psychotic disorders [7,385].

Thus, despite some reports linking BoDV-1 infection with schizophrenia, others have not established a definitive association. For example, a study evaluating BoDV RNA in peripheral blood mononuclear cells from individuals with schizophrenia in the first episode and matched controls reported no causal relationship [386]. Meanwhile, other molecular investigations that use PCR assays targeting BoDV have failed to detect viral nucleic acids in brain, CSF, and blood samples from schizophrenic and control cohorts [306]. In addition, similar findings were echoed in an Iranian cohort study that examined BoDV p40 RNA in individuals with schizophrenia or bipolar disorder, where no significant association with psychiatric pathology was identified [387]. However, conflicting findings persist, as several well-designed studies failed to replicate these associations [368,377,388,389].

Various downstream pathophysiological mechanisms have been implicated in BoDV infection, encompassing viral entry pathways, oxidative stress, neurodegeneration, neuroregeneration, neuroprotection, direct viral cytotoxicity, inflammatory toxicity, and inflammation-driven alterations in neurotransmitter systems [34]. Possibly transmitted via the olfactory system, BoDV utilizes axonal transport to spread through the central nervous system, where it disrupts cytokine signalling, neurotrophin balance, and apoptotic pathways [117].

Regarding the potential mechanisms implicated in the association between BoDV and schizophrenia, mechanistic studies have demonstrated that BoDV-1-infected astrocytes activate microglia, resulting in enhanced levels of inflammatory mediators including MHC I/II, IL-6, TNF-α, and IL-1, which are also implicated as potential biomarkers in schizophrenia pathogenesis [390]. On the other hand, pro-inflammatory cytokines produced during maternal viral infection, particularly IL-6, are known to modulate neurodevelopmental processes, including cell proliferation, neurite extension, and synaptic protein expression, factors that may be aberrant in schizophrenia [360]. Moreover, elevated serum levels of IL-6 have been detected in individuals with schizophrenia and schizoaffective disorder, together with their first-degree family members with mood disorders, suggesting an activated inflammatory response possibly driven by viral agents [18].

Therefore, BoDV infection can cause sustained immune responses, indirectly contributing to central nervous system injury [278]. Moreover, experimental data indicate that BoDV can infect brain tissue and induce behavioural alterations even in the absence of classical inflammatory responses [374].

Therefore, BoDV may be among the heterogeneous group of environmental risk factors that interact with genetic predispositions to influence synaptic plasticity and cortical microcircuit development, contributing to the pathophysiology of psychotic disorders [391].

In conclusion, the role of BoDV remains one of the most debated topics in neurovirology, with most evidence suggesting that BoDV-1 infection alone does not directly induce psychiatric disorders, although it can contribute to their development under certain conditions [382,383,384].

## 14. Human Coronavirus Infection and SARS-CoV-2 (COVID-19)

**Figure 6:**
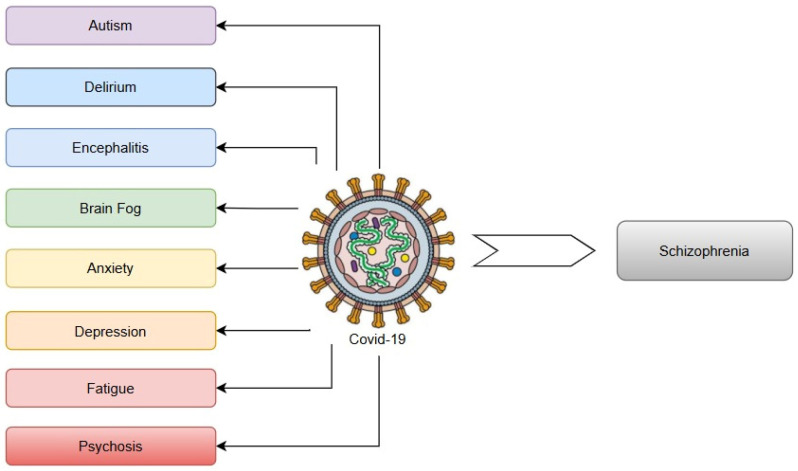
Neuropsychiatric sequelae of COVID-19.

Although primarily characterised as a respiratory illness, COVID-19 (coronavirus disease 2019) has demonstrated significant multisystemic involvement, including effects on the central nervous system (see Figure 6). Coronaviruses, including SARS-CoV-2, exhibit neuroinvasive potential, entering the brain via the olfactory pathway, with the viral presence confirmed in postmortem brain tissue [392].

Moreover, mother-to-child transmission of the virus has been proposed, with reports indicating the possibility of transplacental passage that may interfere with foetal neurodevelopment [392]. It has been hypothesised, though not yet conclusively demonstrated, that offspring of mothers who contracted SARS-CoV-2 may face an elevated probability of neurodevelopmental disorders, particularly those within the autism and schizophrenia spectrum [393]. As a result, it has been theorised that individuals infected with COVID-19 may experience a higher incidence of such disorders in the coming decades [351]. Meanwhile, elevated concentrations of IL-6 have been consistently documented in individuals with COVID-19, including during gestation, and function as an indicator of maternal immune activation, a process that has been implicated in the developmental origins of schizophrenia in offspring. Maternal immune activation during COVID-19, driven by elevated IL-6, IL-8, TNF-α, and other pro-inflammatory mediators such as IL-1β, disrupts foetal neurodevelopment by priming microglia, altering dopaminergic/γ-aminobutyric acidergic (GABAergic) pathways, and activating complement cascades. Systemic inflammation, triggered by SARS-CoV-2, increases placental permeability, allowing cytokines to cross and impair critical processes like neuronal migration and synaptic pruning. These perturbations, particularly during early gestation, heighten vulnerability to schizophrenia by creating a neuroinflammatory substrate that interacts with genetic and later environmental stressors [394]. In particular, deficit schizophrenia, a subtype marked by primary and persistent negative symptoms, has been more strongly correlated with increased levels of pro-inflammatory mediators including IL-6 and TNF- α compared to non-deficit forms. This immunological distinction may be relevant to understanding the interplay between schizophrenia and the progression or outcomes of COVID-19 infection [395].

Furthermore, an increasing spectrum of neuropathological outcomes related to SARS-CoV-2 has been identified to be prevalent among hospitalised patients, reported to affect approximately one-third of this population [396]. Studies have shown numerous neuropsychiatric complications associated with COVID-19, such as delirium, cerebrovascular events, and encephalitis [394]. Interestingly, one of the earliest and most frequently documented symptoms of COVID-19 is impairment of gustatory and olfactory functions. Given that the olfactory pathway is anatomically linked to the temporal lobes, it is not surprising that instances of psychosis, characterised by symptoms commonly associated with temporal lobe activity, have been increasingly reported [397].

Additionally, a frequently reported symptom of post-acute neuropsychiatric COVID-19 syndrome is the phenomenon commonly referred to as ‘brain fog’, which affects approximately 9–55% of individuals for several months after COVID-19 infection. This condition is characterised by impairments in attention, memory, and cognitive endurance, and is believed to be mediated by underlying immuno-inflammatory processes that contribute to reduced executive functioning [398]. A subset of individuals recovering from COVID-19, often referred to as ‘long-haulers’, have reported enduring neuropsychiatric manifestations that persist for several months after infection. In addition to ‘brain fog’, these symptoms encompass fatigue, anxiety, depressive states, and psychotic episodes. In particular, such prolonged neuropsychiatric effects have been observed even among individuals with initially mild presentations of the disease, with approximately 10% of all patients estimated to experience persistent symptoms [394]. On the other hand, the likelihood of developing psychiatric sequelae within 6 months after SARS-CoV-2 infection was higher than that observed after other pulmonary illnesses [399]. These findings underscore the capacity of COVID-19 to disrupt brain function, cognition, and behaviour, highlighting the need for a complete understanding of its neuropathological effects [392].

Some human coronaviruses (HCoVs) have been associated with the onset of psychotic disorders. For example, SARS, a coronavirus closely related to COVID-19, has been documented to precipitate episodes of acute psychosis [400]. Another study that evaluated immunoglobulin G responses to four human coronavirus strains (HKU1, OC43, NL63, and 229E) in individuals with recent-onset psychotic manifestations, relative to control subjects without mental illness, revealed a significant association between the NL63 strain and schizophrenia-spectrum disorders [6].

Furthermore, numerous individuals infected with the virus during the SARS-CoV-2 pandemic presented symptoms of psychosis [401]. As a result, schizophrenia should be recognised as a potential delayed effect of this infection, given the documented association between viral infection and the onset of this psychiatric disorder [402,403,404]. Thus, recent research has revealed that critical cases of SARS-CoV-2 are linked to an elevated risk of developing schizophrenia of 11% [402]. Moreover, a separate study has indicated that the incidence of newly diagnosed schizophrenia among elderly individuals rose during the SARS-CoV-2 pandemic, aligning with existing evidence that the virus poses a higher fatality risk for this population [405]. Other studies, primarily case reports, have explored distinct presentations of psychosis potentially associated with COVID-19 infection or pandemic-related psychological stress. Notably, the average age of affected individuals, 43.9 years, was higher than what is typically observed in cases of schizophrenia of the first episode [406]. Varatharaj et al. conducted a study in the United Kingdom that examined neuropsychiatric manifestations within a COVID-19 patient database and identified cases of newly emerging psychosis. Among the 125 individuals included in the analysis, 23 met established clinical criteria for psychiatric diagnoses. Notably, 10 of these 23 patients (43%) who exhibited neuropsychiatric conditions were found to have new-onset psychosis [407].

Emerging evidence also indicates that the association between COVID-19 infection and psychiatric conditions, such as schizophrenia, might be facilitated by the involvement of the host defence system [401,403,404]. Viral infection has the ability to trigger an abnormal immune reaction, culminating in neuroinflammation, which may represent a plausible mechanism underlying the onset of psychosis [401,403]. Therefore, it is known that increased concentrations of cytokines, found in both COVID-19 and psychiatric conditions, can interfere with brain function by affecting neurotransmitters such as dopamine, serotonin, and norepinephrine. These immune-related changes can also disrupt metabolism and contribute to behavioural symptoms [403]. On the other hand, the hematogenous pathway of viral entry can directly target brain microvascular endothelial cells, which comprise the blood–brain barrier, contributing significantly to the initiation of neural injury [408].

Additionally, the probable involvement of COVID-19 infection in the pathophysiology of schizophrenia can be elucidated through various molecular biomarkers activated by this virus. For example, IL-6, a cytokine implicated in the aetiology of schizophrenia, is also a key mediator of the characteristic cytokine storm of COVID-19 infection [409,410]. Moreover, multiple other molecular markers linked to schizophrenia have been found to exhibit increased expression in response to SARS-CoV-2 infection. These include Apolipoprotein L2 (APOL2), Apolipoprotein L4 (APOL4), Chitinase 3-like 1 (CHI3L1), Synapsin II (SYN2), and methylenetetrahydrofolate reductase (MTHFR) [410].

It has also been observed that angiotensin-converting enzyme 2 (ACE2), the host receptor mediating the entry of both SARS-CoV and SARS-CoV-2, exhibits relatively elevated expression in cerebral areas engaged in schizophrenia, including glutamatergic, dopaminergic, and serotonergic nuclei, the lateral ventricles, and the substantia nigra, despite its generally low expression in the central nervous system. ACE2 is also genetically coexpressed with dopamine decarboxylase, an enzyme implicated in the synthesis of dopamine and serotonin, which are key neurotransmitters implicated in schizophrenia. Given that SARS-CoV has been shown to downregulate ACE2, it is hypothesised that SARS-CoV-2 may similarly disrupt neurotransmitter homeostasis, potentially contributing to the development or exacerbation of psychotic symptoms [394].

Another plausible elucidation for the correlation between coronavirus infection and the emergence of psychosis-like symptoms lies in the ability of the virus, comparable to that of other viruses including influenza, CMV, and HSV, to enhance the expression of Toll-like receptor (TLR) mRNA. Subsequently, this enhancement contributes to an increased production of interferons and cytokines. Elevated concentrations of these cytokines and chemokines can traverse the placenta and reach the foetus, potentially inducing alterations in the central nervous system, and leading to atypical neurodevelopment [411]. Furthermore, maternal infection with COVID-19 can be linked to prenatal and perinatal complications, including pre-eclampsia and premature delivery, both of which are recognised as potential triggers for the onset of psychotic disorders [412].

On the contrary, during the current outbreak of the novel coronavirus disease, it has been proposed that such infections may be linked to disruptions in the peripheral olfactory system, which could subsequently lead to impairments in higher-order brain functions associated with psychotic manifestations [413]. Therefore, viral infections such as SARS-CoV-2 have been shown to damage the olfactory epithelium, which is adjacent to the olfactory bulb and is linked through direct neuronal pathways. Nonetheless, the extent to which alterations in the olfactory epithelium can influence the olfactory bulb within the pathophysiological framework of psychotic disorders remains unclear [414]. It has been proposed that olfactory ensheathing glial cells, located within the olfactory nerve and olfactory bulb, can function as intermediaries between ACE2-expressing cells in the nasal olfactory lining and its sensory neuronal components, thereby initiating a localised immunological reaction. Given that microglia within the olfactory bulb exist in an inherently activated condition, their stimulation may precipitate a chain reaction of cytokine activation, potentially leading to broader neuroinflammatory processes [394]. On the other hand, the olfactory impairment observed in rodent models has been correlated with the emergence of behavioural alterations [413].

While SARS-CoV-2 has been shown to induce inflammation in the olfactory epithelium that may facilitate central nervous system entry [397], the olfactory pathway has also been demonstrated to serve as a route for central nervous system invasion by several other neurotropic viruses including HSV-1, HHV-6, and BoDV [112,117,220,397,413]. Additional studies indicate that rabies virus, vesicular stomatitis virus (VSV), mouse hepatitis virus (MHV), and certain influenza strains can similarly exploit this pathway to reach the brain. These viruses employ distinct mechanisms, either through direct neuronal transport (as seen with rabies and VSV) or by inducing local inflammation that compromises the olfactory epithelium barrier (characteristic of influenza infection) [112,117].

Consistent olfactory dysfunction has also been documented in individuals with psychosis, including those experiencing a first episode of psychosis, with morphological alterations observed in the olfactory bulb, a critical component of the peripheral olfactory structures. Moreover, impairments in olfactory function have been shown to be predictive of unfavourable clinical outcomes in individuals with schizophrenia and may serve as markers to identify those at elevated risk of persistent negative symptoms, notably anhedonia [414]. In the clinical context, an increase in the severity of negative clinical signs has been observed in individuals with schizophrenia during the COVID-19 pandemic [412].

In addition to those biological consequences of SARS-CoV-2 that may elevate the likelihood of schizophrenia onset (see Table 4), the pandemic itself appears to contribute to a rise in reactive psychotic disorders. This phenomenon can be interpreted through the lens of the diathesis–stress model. According to this framework, psychosocial stressors may pathologically activate microglial cells, potentially leading to excessive synaptic pruning and a subsequent reduction in cortical grey matter volume. Such neuroanatomical alterations in stress-sensitive regions may cause immediate cognitive impairments, particularly negative symptoms. Furthermore, diminished cortical regulation could impair the modulation of subcortical dopaminergic activity, thereby facilitating the emergence of positive psychotic symptoms [404].

## 15. Other Viruses Associated with Schizophrenia

The extensive range of viral agents associated with schizophrenia suggests that the fundamental cause is unlikely to be specific to any single pathogen but rather reflects an immune-mediated process. Additionally, research suggests that certain viruses may disrupt dopaminergic pathways and modulate glutamatergic signalling through molecular mimicry at NMDA receptors, potentially contributing to the emergence of psychotic features [394].

**Figure 7:**
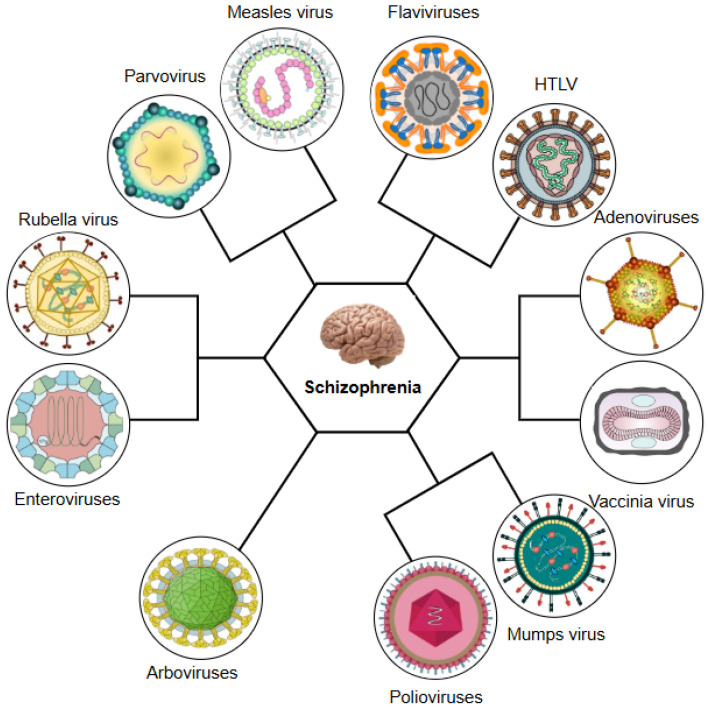
Other viral contributors to schizophrenia. HTLV: human T cell leukaemia virus.

In addition to the viruses discussed above, various other viral pathogens have been involved in the development of schizophrenia (see Figure 7). These include parvovirus B19, measles virus, rubella virus, enteroviruses including Coxsackie B4 and B5, arboviruses comprising Eastern equine encephalitis virus, poliovirus, mumps virus, vaccinia virus, and various retroviruses [16,18,36,415,416,417,418,419,420]. Adenoviruses have also been identified in elderly patients diagnosed with schizophrenia, suggesting potential viral involvement in the disorder’s aetiology [17] (see Figure 7).

Viruses belonging to the Flaviviridae family, including Powassan virus, West Nile virus (WNV), and dengue virus, have also been correlated with the manifestation of psychotic symptomatology [421]. Increasing evidence indicates associations between flaviviral pathophysiological mechanisms, immune responses, and the emergence of psychotic disorders. The geographical distribution of schizophrenia has been correlated with the prevalence of tick-borne encephalitis, including infections caused by tick-associated flaviviruses. Experimental research in murine models has demonstrated that certain genomic elements of flaviviruses, including the Kunjin subtype of WNV, exhibit homology with aberrant protein-coding sequences identified in individuals with schizophrenia [21]. Additionally, encephalitis induced by RNA viruses has been implicated in cerebellar neuroinflammation, a neuropathological condition increasingly recognised in both clinical and preclinical studies as associated with psychotic features [421]. Furthermore, with regard to dengue fever, empirical studies have identified its potential to precipitate a range of psychiatric manifestations, including manic episodes, anxiety, depressive symptoms, catatonia, and, in some instances, acute psychotic episodes [422,423].

Furthermore, HTLV, a retrovirus, has been shown to multiply within central nervous system cells, thereby inducing neurological and psychiatric manifestations within a limited group of infected subjects [18]. In particular, a clinical trial conducted in Brazil reported a high prevalence of psychiatric disturbances among individuals infected with HTLV-1, pointing to a possible link between this infection and psychiatric conditions [424].

Some epidemiological investigations have proposed that infection with parvovirus B19, a TORCH agent known for its capacity to cross the placental barrier and infect the foetus, may lead to schizophrenia. However, this theory remains preliminary and requires validation through large-scale longitudinal studies [415,425]. In particular, investigations using animal models have demonstrated that neurodevelopmental impairments may manifest postnatally following intrauterine infection with parvoviruses. These delays in neurological and behavioural development resemble patterns observed in pervasive developmental disorders such as autism and may extend to schizophrenia and bipolar disorder. Furthermore, the seasonal distribution of parvovirus B19 infections, which occur predominantly during winter and early spring, is correlated with epidemiological findings that indicate an approximately 10% increase in schizophrenia births during late winter and early spring. Moreover, a molecular study involving 104 human dorsolateral prefrontal cortex DNA samples was conducted to assess the presence of parvoviral DNA, specifically targeting adeno-associated virus 2 (AAV2) and B19. The results concluded that such viruses are capable of infecting and persisting in brain tissue. This persistence may enable interactions with host genetic susceptibilities, which may influence the susceptibility of the disease in high-risk populations [415].

Another virus potentially involved in the mechanisms of schizophrenia is the measles virus, a virus with an RNA genome and affinity for neural tissue, classified under the Paramyxoviridae family, which has demonstrated the capacity to persist within the central nervous system [11,417,426]. Of particular historical relevance is the identification, between 1980 and 1982, of virus-like particles that resemble paramyxoviral nucleocapsid aggregates in brain cortex samples from five patients diagnosed with febrile schizophrenia, suggesting a possible viral contribution to pathogenesis [427].

The progression and outcome of measles virus infection appear to be influenced by host-related genetic factors. In this context, it is particularly noteworthy that human leukocyte antigen (HLA) molecules hold a central position in modulating the immunological reaction to the measles virus, and the genes encoding these molecules have been linked to enhanced susceptibility to schizophrenia in genome-wide association studies [417]. Furthermore, increased levels of measles-specific antibodies have been identified in patients with newly emerged psychotic disorder relative to those with chronic schizophrenia. These concentrations were also substantially raised in individuals with psychosis compared to non-psychiatric controls, suggesting that antibody concentrations may peak during the early course of the illness [417,428,429]. In addition, some patients diagnosed with subacute sclerosing panencephalitis, an uncommon delayed neurological complication related to measles, exhibit psychotic features and other neuropsychiatric manifestations that partially overlap with schizophrenia [417]. Psychotic symptoms have also been observed sporadically in subjects after recent measles virus infection [430]. Moreover, increased intrathecal antibody concentrations targeting the measles virus have been identified in a subset of individuals with schizophrenia [426]. Additional epidemiological evidence was provided by Torrey et al., who identified significant associations between prenatal exposure to measles and VZV and increased risk of schizophrenia, particularly during the fifth to seventh months of gestation [129].

Global poliomyelitis epidemics provide another opportunity to examine the potential association between congenital viral exposure and the subsequent vulnerability of developing psychotic conditions. Poliovirus was initially suggested as a possible aetiological factor for schizophrenia following observations of a decline in schizophrenia incidence in multiple countries after the widespread implementation of the poliomyelitis vaccine [418].

In Finland, a population-based analysis demonstrated that prenatal contact with poliovirus, specifically around five months before birth, was correlated with an enhanced probability of schizophrenia in adulthood. This finding aligns with a substantial body of research suggesting that infectious insults during the middle trimester of pregnancy may compromise neurodevelopmental outcomes [431]. An attempt to replicate these findings was undertaken in Australia and New Zealand, where researchers analysed the impact of eight well-defined outbreaks of poliomyelitis in Queensland. However, this research did not demonstrate a statistically meaningful correlation between foetal exposure to poliovirus and the subsequent vulnerability of schizophrenia [432]. Of particular interest is the temporal correlation observed between the eradication of poliomyelitis and the near vanishing of catatonic schizophrenia among population cohorts born after the elimination of polio. This remarkable pattern suggests a possible causal relationship between prenatal or early postnatal poliovirus exposure and the subsequent appearance of catatonic schizophrenia. Consistent with this, the cumulative incidence of catatonic schizophrenia in birth cohorts from 1956 to 1989 declined by over 90%. Given that catatonic schizophrenia typically presents with an earlier age of onset, a reduction in poliovirus circulation may help explain the observed decline in early-onset schizophrenia cases [433].

On the contrary, rubella has considerable clinical relevance due to its well-documented capacity to directly invade the foetal brain, inducing atypical neurodevelopmental processes such as neuronal apoptosis, gliosis, and mitotic disruption [434]. Furthermore, magnetic resonance imaging findings have revealed that individuals with congenital rubella who exhibit schizophrenia-spectrum manifestations have diminished cortical grey matter and enlarged lateral ventricles, neuromorphological anomalies that are frequently found in individuals diagnosed with schizophrenia [435].

In a landmark investigation, Brown et al. [434] longitudinally assessed a birth cohort in which intrauterine exposure to rubella was confirmed through serological testing and clinical evaluation during the 1964–1965 epidemic. This cohort was compared with two control groups born after 1967, a period during which the incidence of rubella was minimal, thus rendering prenatal exposure improbable. The findings indicated a significantly elevated prevalence of non-affective psychotic disorders among rubella-exposed individuals (15.7%) relative to the unexposed counterparts (3.0%).

Among the most compelling empirical data supporting the association between prenatal viral infections and schizophrenia emerges from the Rubella Birth Defects Evaluation Project. In this sample population, characterised by serologically verified prenatal rubella exposure, the incidence of schizophrenia and related spectrum disorders exceeded 20%, implying a ten- to fifteenfold elevation in risk. Additionally, a pronounced decline in intellectual functioning from childhood to adolescence was observed more frequently in the rubella exposed group relative to exposed controls, further substantiating the credibility of the association [420]. Subsequent research has reinforced this linkage, demonstrating that prenatal rubella exposure correlates with cognitive decline that extends into adulthood, as well as with early premorbid behavioural anomalies [420]. However, the results of a separate longitudinal investigation conducted by Buka et al. [51], which monitored over 55,000 pregnancies in the USA from 1959 to 1966 and assessed maternal IgA and IgG antibody titres to rubella, did not reveal a statistically significant correlation between prenatal rubella exposure and the likelihood of schizophrenia in adult progeny.

Type B coxsackieviruses (CV-B), which frequently invade the central nervous system and are documented to trigger neurological conditions including meningitis and encephalitis, have also been investigated for their potential role in neuropsychiatric disorders. Experimental data suggest a possible correlation between CV-B infection and idiopathic neurodegenerative disorders, including amyotrophic lateral sclerosis, and mental disorders including schizophrenia [4].

The underlying processes are thought to involve the host immune response following viral infection, which can contribute to neuropathological changes. Furthermore, the capacity of CV-B to reach and remain in the central nervous system, especially through congenital transmission, and to specifically target neural stem cells underscores the potential for long-term disruptions in neural function [4]. Moreover, inflammatory molecules, including cytokines and chemokines, may disrupt the stability of the blood–brain barrier, which usually preserves the brain’s immune-protected environment. Since blood–brain barrier dysfunction is considered a trait marker for schizophrenia, CV-B-induced neuroinflammation can contribute to this pathological process by allowing peripheral immune elements to alter synaptic and neurotransmitter function [436]. Additionally, during the prenatal and perinatal periods, critical neurodevelopmental windows, CV-B infections have been associated with aberrant cytokine production, including elevated levels of type I interferons, TNF-α, IL-1β, IL-6, MCP-1, and RANTES (Regulated upon Activation, Normal T cell Expressed and Secreted). These immune disturbances can interfere with normal brain maturation, which can lead to the structural and functional abnormalities observed in schizophrenia [4]. Although a definitive aetiological link between CV-B and schizophrenia has not been conclusively established, two prospective cohort studies from Finland reported a higher risk of adult-onset schizophrenia following CV-B-related central nervous system infections during infancy or early childhood. These studies reported odds ratios of 4.8 (follow-up through 1994) [437] and 2.5 (follow-up through 1997) [438].

These results reinforce the theory that CV-B infections during early developmental stages may act as significant determinants in the onset of schizophrenia. Nevertheless, a key limitation of current investigations is the frequently asymptomatic or mildly symptomatic nature of CV-B infections, which may result in underdiagnosis and complicate efforts to establish clear causal relationships [436].

## 16. Conclusions

The evidence reviewed here underscores the growing recognition of the role viral infections, particularly during neurodevelopmentally sensitive periods, may play in the complex pathophysiology of schizophrenia. Although the disorder remains a clinically and biologically heterogeneous syndrome, converging findings suggest that immune dysregulation, low-level neuroinflammation, and persistent viral or postviral activity can contribute significantly to a subset of schizophrenia cases, particularly those resistant to conventional therapy.

Importantly, the mild encephalitis hypothesis offers a unifying framework that integrates infectious, autoimmune, and inflammatory processes, which could explain the overlap of the symptom’s domains of psychiatric disorders. Viral agents can act as initial insults or “late hits” in genetically predisposed individuals, triggering pleiotropic mechanisms that alter neurodevelopment, immune homeostasis, and neurotransmission, particularly by disrupting the blood–brain barrier, cerebrospinal fluid signalling, and volume transmission dynamics. Such pathophysiological processes can be amplified by stress, hormonal changes, and metabolic factors, further complicating the clinical course.

Despite compelling associations, the identification of specific viral aetiologies in schizophrenia remains elusive, largely due to the transient nature of infections, subclinical presentations, and limitations in current neuropathological and diagnostic techniques. However, advances in viromics, such as Virobiome-Seq, offer promising tools for characterising the human virome and its interactions with host immunity and neurobiology. Similarly, genome-wide association studies in conjunction with hypothesis-driven mechanistic research on transposable elements, including HERVs, could provide new insights into how viral and genetic factors converge to influence psychiatric vulnerability.

Prospective epidemiological studies, particularly those investigating prenatal exposure to viruses such as ZIKV and SARS-CoV-2, will be crucial to elucidating the long-term neuropsychiatric sequelae of congenital and perinatal infections. Longitudinal birth cohorts with detailed virological, immunological, and neurocognitive data are needed to trace developmental trajectories and identify early markers of disease risk. In addition, research on viruses with known neurotropic potential, such as EBV, CMV, and HSV, should continue, especially in the context of gene–environment interactions, aberrant immune responses, and their role in the transition to psychosis.

Therapeutically, the potential for the repurposing of antipsychotics with anti-inflammatory and antiviral properties warrants further exploration, particularly for the subgroup of patients exhibiting signs of persistent neuroinflammation. On the other hand, personalised treatment strategies that integrate immune profiling, viral serostatus, and genetic susceptibility can improve outcomes and enable early intervention in high-risk individuals. Furthermore, biomarker discovery, including multiplex assays for cytokines, antibodies, and viral antigens, will be essential for stratifying patients and tailoring therapeutic approaches.

Ultimately, schizophrenia is unlikely to be attributable to a single viral cause. Instead, a multifactorial model that incorporates viral exposures, immune dysfunction, genetic liability, and environmental stressors offers the most plausible framework for understanding their diverse presentations. To address persisting knowledge gaps, we recommend future systematic reviews and meta-analyses evaluating the candidate viruses identified here as potential environmental contributors to schizophrenia-spectrum disorders. Therefore, future research should aim not only to clarify causal pathways but also to harness this knowledge to develop preventive strategies, novel treatments, and precision medicine applications in psychiatry.

## Figures and Tables

**Table 1:** HSV-1 versus HSV-2: comparative influence in schizophrenia.

Feature	HSV-1	HSV-2	References
Epidemiological Association with Schizophrenia	Stronger and more consistent association with schizophrenia risk and progression	Mixed findings; weaker and less consistent association	[37,38,39,40,41,44]
Primary Transmission Route	Oral (via saliva, close contact)	Sexual (genital)	[50,65]
Neuronal Tropism	Preferential infection of the trigeminal ganglia; tropism for frontal/temporal cortices, hippocampus, and limbic system	Lumbosacral ganglia; less neurotropic than HSV-1	[47,50,55,58,59]
Mechanisms of Neuropathology	Direct neuronal damage, chronic neuroinflammation, oxidative stress, mitochondrial dysfunction, apoptosis, and disruption of the NMDA receptor	Primarily immune-mediated (maternal immune activation during pregnancy)	[37,40,41,44,61,62,63,64,66]
Cognitive and Symptomatic Effects	Strongly linked to cognitive deficits (working memory, executive function), negative symptoms, and progressive cortical atrophy	Less clear; possible association with accelerated cortical thinning, the neuroanatomical substrate for cognitive decline, when combined with other infections (e.g., *C. pneumoniae*).	[37,44,46,53,67,68,69,70,71]
Neuroimaging Findings	Diminished grey matter observed in the prefrontal and anterior cingulate cortices, as well as the hippocampus; progressive posterior cingulate atrophy	Cortical thinning and reduction in hippocampal volume (when combined with other infections)	[37,44,53,72,73]
Immunological Impact	Activates neuroinflammation via TLR signalling pathways, involving cytokines including IL-6, IL-1β, and TNF-α, leading to microglial activation	Maternal IgG/IgM antibodies may disrupt foetal neurodevelopment through immune dysregulation	[41,49,51,73,74,75]
Genetic Interactions	Overlap with schizophrenia genome-wide association studies loci (e.g., *NRP1*, HLA alleles); *MICB* polymorphism modifies risk	Gene–environment interactions (e.g., *GRIN2B* variants) in offspring of HSV-2-positive mothers	[39,40,76,77,78]
Therapeutic Implications	Antivirals (e.g., valacyclovir) show modest cognitive benefits in trials; immunomodulation strategies under investigation	Limited evidence; potential focus on maternal–foetal interventions	[41,48,63]

**Table 2:** Pathogenic mechanisms linking EBV, HSV-1, and CMV infection to schizophrenia.

No.	EBV	HSV-1	CMV
1	Genetic predisposition to developing neuropsychiatric effects following EBV infection [79]	Direct neuronal damage via hippocampal and frontotemporal tropism, disrupting neurodevelopment and synaptic function [37,47,58,59]	Congenital infection causing neurodevelopmental disruptions (microcephaly, polymicrogyria) [80,81,82]
2	Impairment of neuronal survival and function through microglial priming, leading to a dysregulated response to subsequent infections [83]	Chronic neuroinflammation triggered by microglial activation (TLR2/3/9) and elevated pro-inflammatory cytokines (IL-6, TNF-α, IL-1β) [41,75,84]	Immune dysregulation through chronic inflammation (elevated TNF-α, IL-10) [16,85,86]
3	Transactivation of endogenous retroviruses by EBV, influencing the regulation of host gene expression [87]	NMDA receptor dysfunction exacerbating glutamatergic hypofunction [66,88]	Neural progenitor cell infection disrupting proliferation/migration [16,80,89,90]
4	Indirect effects via activation of systemic cytokine responses and stress-related pathways, resulting in neuroinflammatory processes within the brain [91,92]	Epigenetic dysregulation in neural progenitor cells (e.g., viral thymidine kinase-mediated interference with differentiation) [60,93]	Latency/reactivation in neural cells causing neuroinflammation [90,94]
5	Modulation of the inflammatory response following EBV infection by immune-related genes, resulting in altered brain development and function [83]	Structural brain abnormalities (grey matter loss in prefrontal cortex, anterior cingulate, and hippocampus) correlating with cognitive deficits [37,44,72,73]	Molecular mimicry triggering autoimmune responses (e.g., against GAD) [95]
6	Dysregulated immune responses to EBV infection [79]	Disrupted neurodevelopmental pathways via nectin-1 receptor-mediated adhesion and connectivity impairments [59,60]	Hippocampal volume reduction and dentate gyrus abnormalities [80,96,97]
7	Disruptions in neurotransmitter receptor interactions [92]	Maternal immune activation (elevated IL-18, CRP) during pregnancy, altering foetal brain development [41,74,98]	Genetic susceptibility via immune-related polymorphisms (TNF-α, IL-10, C4, *CTNNA3*) [16,99,100]
8	The interaction between complement receptor 2, used by EBV for cellular entry, and complement component 4 (C4), which participates in viral inactivation and is also implicated in schizophrenia via allelic variations in the *C4* gene [101]	Genetic interactions (e.g., HLA alleles, *MICB rs1051788*, Fyn kinase) modulating infection susceptibility and neuroinflammation [39,76,77,102]	
9		Latent central nervous system infection with periodic reactivation, causing cumulative neuronal damage and oxidative stress [44,63,103,104]	

**Table 3:** HERV-related mechanisms in schizophrenia pathogenesis.

No.	Mechanism	Description of the Mechanism	References
1	Genetic predisposition	- HERV-K elements may enhance *PRODH*, a schizophrenia susceptibility gene involved in glutamatergic transmission. - The 22q13 region harbours *APOBEC3G*, an inhibitor of retroviral replication, and may represent a potential locus of genetic vulnerability to HERV activation.	[318,320,321]
2	Epigenetic dysregulation	- Hypomethylation of HERV-W and HERV-K loci. - Chromatin remodelling at long terminal repeats (LTRs) facilitating transcriptional activation.	[317,318,319,320]
3	Gene–environment interaction	*Toxoplasma gondii* and viral infections (e.g., influenza, HSV-1, HIV, EBV) can transcriptionally activate HERVs, particularly HERV-W, through inflammatory signalling pathways (e.g., NF-κB) or through direct activation by viral proteins such as Tat or Tax.	[311,316,324,332,333]
4	Neuroimmune mechanisms and neuroinflammation	- Upregulation of inflammatory mediators including TNF-α, IL-6, and IL-1β via NF-κB and JAK-STAT1 signalling pathways, which are triggered by the envelope protein (env) of HERV-W, a potent activator of TLR4. - Induction of oxidative stress and nitric oxide-mediated neurotoxicity via enhanced expression of iNOS, which is triggered by HERV-W Env. - Disruption of the blood–brain barrier. - Microglial activation.	[9,307,322,324,325,329]
5	Neurodevelopmental and neurotransmission disruption	- Disruption of synaptic development, neuronal differentiation, and dopaminergic signalling through HERV-W Env, which has been demonstrated to modulate the expression of critical neuroplasticity genes associated with these processes, such as BDNF, NTRK2/TrkB, and DRD3. - Leading to an excitatory/inhibitive imbalance through the Env protein, which also interacts with sodium-dependent amino acid transporters hASCT1 and hASCT2, disrupting glutamate uptake. - Impaired neuronal excitability and hippocampal long-term potentiation through HERV-W Env, which downregulates 5-HT4 receptors and activates SK2 and SK3 potassium channels.	[308,309,326,327,328,329,330]
6	Ferroptosis	HERV-W ENV downregulates neuroprotective genes such as *GPX4* and *SLC3A2*, resulting in iron accumulation, lipid peroxidation, and mitochondrial dysfunction.	[331]
7	HERV-W activation via NF-κB and viral protein interactions	- HERV-W Env protein binds to the TLR4 receptor, activating NF-κB → releases TNF-α, IL-6, and IL-1β → chronic neuroinflammation and neuronal damage. - Viral protein synergy: HSV-1 (ICP0), HIV (Tat), and EBV (LMP-1) activate NF-κB, which binds to HERV-W promoters → HERV-W transcriptional reactivation. - Epigenetic priming: viral infections cause DNA hypomethylation of HERV-W LTRs, making them responsive to NF-κB → loss of HERV silencing → persistent expression.	[9,307,316,317,319,322,332,333]

**Table 4:** Possible mechanisms linking coronaviruses to schizophrenia.

No.	Mechanism	References
1	Maternal immune activation, which interferes with foetal neurodevelopment.	[392,394]
2	Immune-mediated mechanisms.	[401,403,404]
3	Direct neural damage by targeting the brain’s microvascular endothelial cells.	[408]
4	Triggering molecular markers associated with schizophrenia: interleukin-6 (IL-6), Apolipoprotein L2 (APOL2), Apolipoprotein L4 (APOL4), Chitinase 3-like 1 (CHI3L1), Synapsin II (SYN2), and methylenetetrahydrofolate reductase (MTHFR).	[409,410]
5	Downregulation of angiotensin-converting enzyme 2 (ACE2), leading to disruption of the dopaminergic and serotonergic systems.	[394]
6	Upregulation of Toll-like receptor (TLR) mRNA expression, resulting in elevated levels of cytokines and chemokines.	[411]
7	Disruption of the peripheral olfactory system.	[413]
